# Alternative contingency table measures improve the power and detection of multifactor dimensionality reduction

**DOI:** 10.1186/1471-2105-9-238

**Published:** 2008-05-16

**Authors:** William S Bush, Todd L Edwards, Scott M Dudek, Brett A McKinney, Marylyn D Ritchie

**Affiliations:** 1 Center for Human Genetics Research, Department of Molecular Physiology and Biophysics, Vanderbilt University Medical Center, Nashville, Tennessee, USA; 2 Department of Genetics, University of Alabama School of Medicine, Birmingham, Alabama, USA

## Abstract

**Background:**

Multifactor Dimensionality Reduction (MDR) has been introduced previously as a non-parametric statistical method for detecting gene-gene interactions. MDR performs a dimensional reduction by assigning multi-locus genotypes to either high- or low-risk groups and measuring the percentage of cases and controls incorrectly labelled by this classification – the classification error. The combination of variables that produces the lowest classification error is selected as the best or most fit model. The correctly and incorrectly labelled cases and controls can be expressed as a two-way contingency table. We sought to improve the ability of MDR to detect gene-gene interactions by replacing classification error with a different measure to score model quality.

**Results:**

In this study, we compare the detection and power of MDR using a variety of measures for two-way contingency table analysis. We simulated 40 genetic models, varying the number of disease loci in the model (2 – 5), allele frequencies of the disease loci (.2/.8 or .4/.6) and the broad-sense heritability of the model (.05 – .3). Overall, detection using NMI was 65.36% across all models, and specific detection was 59.4% versus detection using classification error at 62% and specific detection was 52.2%.

**Conclusion:**

Of the 10 measures evaluated, the likelihood ratio and normalized mutual information (NMI) are measures that consistently improve the detection and power of MDR in simulated data over using classification error. These measures also reduce the inclusion of spurious variables in a multi-locus model. Thus, MDR, which has already been demonstrated as a powerful tool for detecting gene-gene interactions, can be improved with the use of alternative fitness functions.

## Background

The statistical definition of epistasis was given by Fisher in 1918 as deviations from additive effects in a linear statistical model [1]. The idea of epistasis, or more generally "gene-gene interaction", has reappeared as a popular theme in human genetics over the last ten years. There is a growing belief that susceptibility to common diseases may be governed by the potentially complex interaction of multiple genetic variants. This belief is driven largely by the notion that large biochemical networks and gene regulatory processes involving multiple genes have a functional endpoint that may be influenced by the simultaneous presence of multiple variants in those genes [2,3].

In addition to its theoretical importance, epistasis has been functionally demonstrated to play a role in human disease. Most notably, Hirschsprung's disease was found to be influenced by polymorphisms in *RET *and the *ERDB2 *receptor in the Old Order Amish and was confirmed in a mouse model [4]. Having both variants simultaneously increases risk of disease far beyond the combined risk of each independent variant.

As epistasis is believed to have important implications for human disease risk, numerous statistical and computational approaches have been developed to examine epistasis in family-based and case-control association studies [5-9]. Multifactor dimensionality reduction (MDR) is one such computational method to identify gene-gene interactions in case-control studies where variants may or may not exhibit detectable marginal effects. MDR has been shown previously to have reasonable power to detect gene-gene interactions in several cases of experimental error and over a variety of simulated genetic models [10]. MDR has also been applied to many disease phenotypes including hypertension [11-13], multiple sclerosis [14], sporadic breast cancer [15], type II diabetes [16], coronary artery disease [17], and autism [18].

There have been several notable extensions to the MDR method. Multifactor Dimensionality Reduction Pedigree Disequilibrium Test (MDR-PDT) was developed by Martin et al. to examine multi-locus models in extended pedigree data [19]. MDR was extended to use a chi-square statistic as an alternative to prediction error/classification error, and to test other forms of cross-validation and permutation testing [20]. MDR was also modified to include the odds ratio as a quantitative measure of disease risk [21] – as well as extended into a generalized MDR (GMDR) to include discrete and quantitative covariates as well as dichotomous and continuous phenotypes [22]. Velez et al. evaluated the performance of MDR using balanced accuracy for several examples of class-imbalance [23]. As the balanced accuracy measure provided improved power for cases of class imbalance, we hypothesized that alternate measures of classification performance would improve the performance of MDR. In this study, we demonstrate through simulated data that altering the scoring measure used in model evaluation and selection can improve the detection and statistical power for complex interaction effects. For the purposes of this study, simulated epistasis models contain no marginal effect, where all phenotypic variance attributable to genetic variation is due completely to the interaction of genetic loci, as described by Culverhouse et al. [24] To detect these genetic effects, the influential loci must be examined jointly.

**Figure 1:**
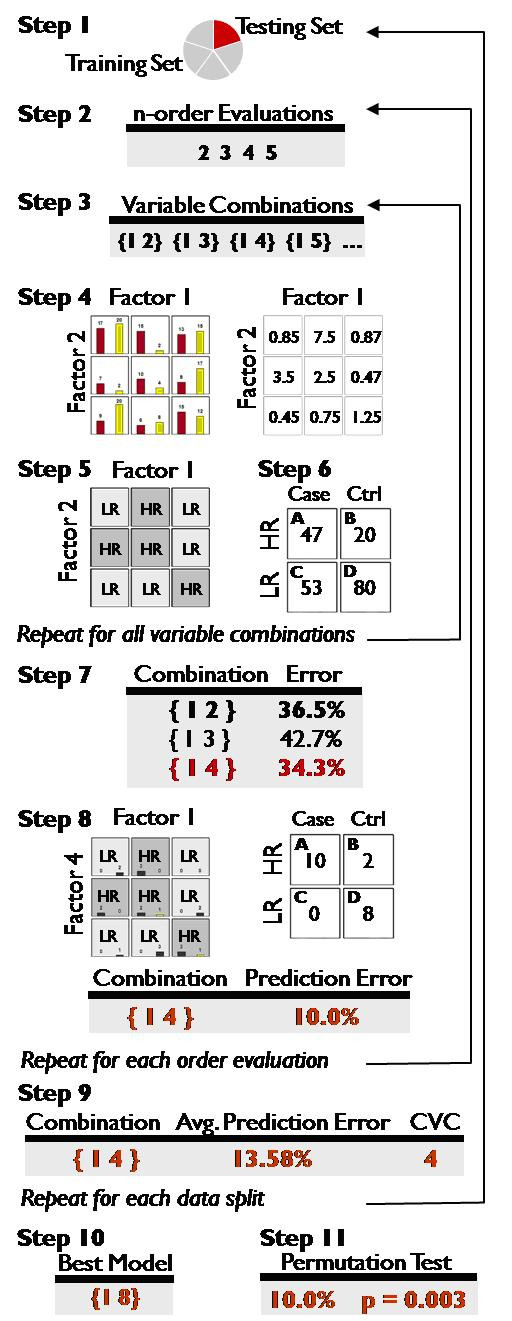
**MDR process**. For a full description of the MDR algorithm, please see the "Methods" section.

MDR is a non-parametric statistical method for the analysis of gene-gene and gene-environment interactions[15,25]. Figure 1 illustrates the MDR algorithm. Additional detail is provided in the "Methods" section. MDR performs an exhaustive search of all N-locus models, collapsing multi-locus genotypes into high-risk and low-risk classes. These high- or low-risk classes are then compared to the observed status of individuals to produce a score for the classification. In this manner, all N-locus models are ranked by a scoring measure, and the model with the optimal score is selected as the best or "most fit."

### Fitness Measures

**Figure 2:**
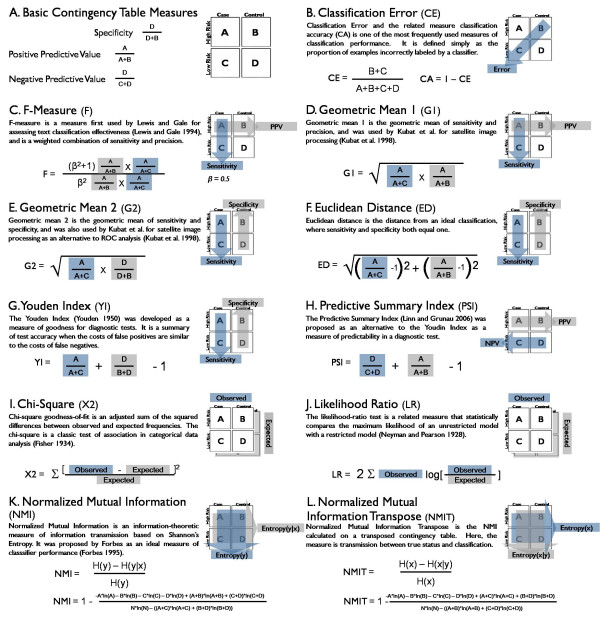
**Contingency table measures of classification performance**. Four basic contingency table measures (A) can be combined to form several composite measures of contingency table fitness (B-L).

The results of a classification algorithm can be represented as a special type of two-way contingency table (also called a confusion matrix). The true status forms one dimension of the table and the algorithm classification forms the other dimension. Two-way tables can be scored with a variety of measures, many of which have been developed in multiple disciplines using different terminology to describe similar concepts. There are several basic terms and measures using the four cells of a two-way contingency table (Figure 2A).

Sensitivity is the classification accuracy of the cases, or the proportion of correctly classified cases among all cases in the data. In the text classification field, this measure is called recall. Specificity is the classification accuracy of the controls, or the proportion of correctly classified controls among all controls in the data. Positive Predictive Value or Precision is the classification accuracy of the affirmative classification, or the proportion of actual cases among all individuals classified as cases. Negative Predictive Value is the proportion of controls among all individuals classified as controls. Using these basic values, several composite measures of association have been developed (Figure 2B–L). The ten measures selected represent a variety of analysis strategies from several fields including text classification, machine learning, diagnostic testing, statistical theory, and information theory. Classification error (CE) and the related quantity, classification accuracy (1-CE), are two of the most frequently used and contentious measures of classification performance. It is defined simply as the proportion of examples incorrectly labelled by a classifier. The technical merits of classification error as a measure of classifier performance have been debated [26-29].

#### Precision-based and ROC-based measures

F-measure (F) (Figure 2D) is a measure first used by Lewis and Gale for assessing text classification effectiveness, and is the inverse of the E-measure [30]. The E-measure is a weighted combination of sensitivity and positive predictive value derived by van Rijsbergen to satisfy several conditions of measurement theory [31]. Geometric means have been used as performance measures for classification. Kubat et al. defines two such quantities, here labelled geometric mean 1 and geometric mean 2 [32]. Geometric mean 1 (G1) (Figure 2C) is the geometric mean of sensitivity and precision. Geometric mean 2 (G2) (Figure 2E) is the geometric mean of sensitivity and specificity. This measure is related to the receiver-operator characteristic (ROC) curve and was used in lieu of ROC analysis by Kubat et al. as a single measure of classification [27,32]. Euclidean distance from an ideal classification (ED) (Figure 2F) is a measure also related to ROC curves. This combination of sensitivity and specificity measures the distance from an ideal classification in ROC space, where sensitivity and specificity both equal one.

#### Diagnostic testing measures

The Youden index (YI) (Figure 2G) and Predictive Summary Index (PSI) (Figure 2H) are summary measures of certainty for dichotomous diagnostic tests [33,34]. The Youden index is the sum of the sensitivity and specificity minus one. The predictive summary index is the sum of the positive predictive value and the negative predictive value minus one.

#### Statistical measures

Pearson's Chi-square goodness-of-fit statistic (χ^2^) (Figure 2I) is an adjusted sum of the squared differences between observed and expected frequencies [35]. The chi-square is a classic test of association in categorical data analysis. The likelihood-ratio test (LR) (Figure 2J) is a related measure that statistically compares the maximum likelihood of an unrestricted model with a restricted model [36]. In this setting, the unrestricted model consists of the observed frequencies in the data and the restricted model consists of the expected frequencies under the null hypothesis of no association.

#### Information theoretic measures

Normalized Mutual Information (NMI) (Figure 2K) was described by Wickens as a measure of information transmission, based on Shannon's Entropy [37]. Entropy was developed in communication theory as a measure of dispersion for categorical data. Entropy is often measured in bits, or log base 2 units. Given a two-way contingency table, four entropy values can be computed: the row entropy, the column entropy, and two conditional entropies (H(x|y)) not shown):

(1) $$H\left(x\right)=-\sum_{i}p_{i}\log_{2}p_{i}$$

(2) $$H\left(y\right)=-\sum_{j}p_{j}\log_{2}p_{j}$$

(3) $$H\left(y|x\right)=\sum_{i}p_{i}\left[-\sum_{j}\frac{p_{ij}}{p_{i}}\log_{2}\frac{p_{ij}}{p_{i}}\right]$$

The quantities p_i _and p_j _represent the empirical probabilities of the predicted and true class states, respectively, and p_ij _is their joint probability. Using these values, NMI and its transpose (NMIT) (Figure 2L) are defined as:

(4) $$NMI\left(y\right)=\frac{H(y)-H(y|x)}{H(y)}$$

(5) $$NMIT\left(y\right)=NMI(x)=\frac{H(x)-H(x|y)}{H(x)}$$

The NMI value is interpreted as the proportion of information contained in the row variable that is transferred or transmitted to the column variable, or more concisely the amount by which the model reduces our uncertainty about the true state.

## Results

### Detection

**Figure 3:**
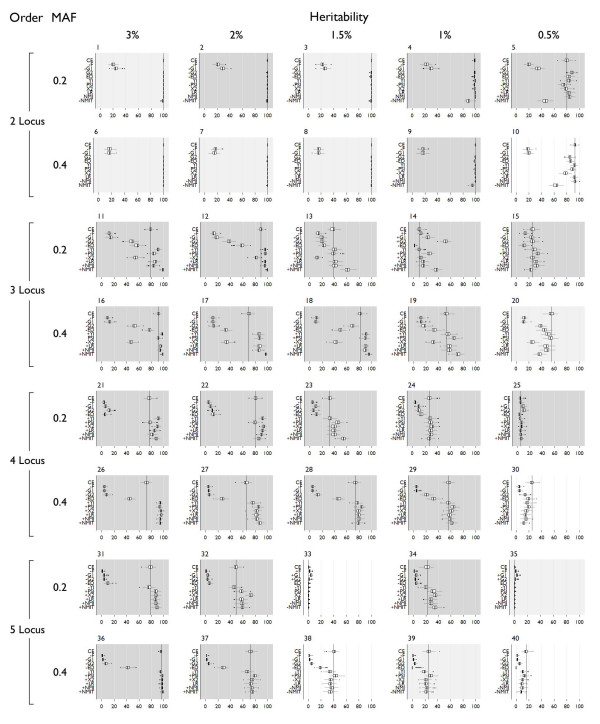
**Detection box plots**. The dark vertical line indicates the mean detection using classification error. Dark shading indicates LR and NMI were significantly better, medium shading indicates no significant difference. Light shading indicates classification error was better than LR and NMI. "+" on the y axis indicates significantly different from classification error with a higher mean, "-" indicates significantly different from classification error with a lower mean. Significance was assigned by Wilcoxon rank-sum tests.

**Figure 4:**
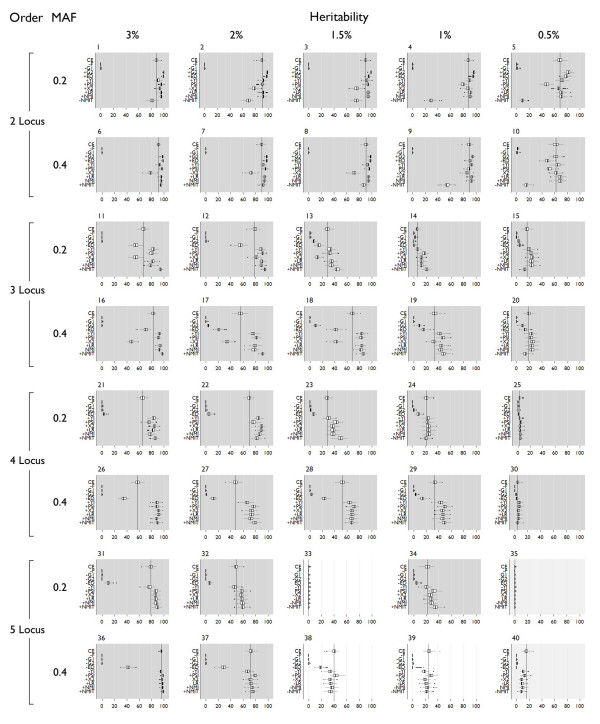
**Specific detection box plots**. The dark vertical line indicates the mean detection using classification error. Dark shading indicates LR and NMI were significantly better, medium shading indicates no significant difference. Light shading indicates classification error was better than LR and NMI. "+" on the y axis indicates significantly different from classification error with a higher mean, "-" indicates significantly different from classification error with a lower mean. Significance was assigned by Wilcoxon rank-sum tests.

All detection results are shown in Figure 3 and specific detection results are in Figure 4. "Detection" is the ability of the method to correctly identify all disease loci, but additional non-disease loci may be included in the model also. "Specific detection" is the ability of the method to correctly identify all the disease loci and no additional loci – this could also be phrased as the ability to detect the correct multi-locus model with no false positive loci. Significant differences from classification error (indicated by "+" and "-" symbols in Figures 3 and 4 to indicate higher mean and lower mean results respectively) had Wilcoxon rank-sum p-values below 0.05. Over all models, the mean detection using classification error was 62% and the mean for specific detection was 52.2%. Detection was at or above 80% in all two-locus models, with 100% detection for all two-locus models with ≥ 1.5% heritability. Detection diminishes in the three-locus models, with only three models showing greater than 80%. Detection in four- and five-locus models drops below 80% except for model 35, a five-locus model with 3% heritability, which was detected at 96%. Specific detection using classification error is above 80% for all two-locus models except the 0.5% heritability models (models 5 and 10). All three-, four- and five-locus models are below 80% specific detection except for model 35, with specific detection at 95%. These models show sporadic detection and specific detection that does not follow trends based on allele frequency or heritability.

The precision-based measures F-measure and geometric Mean 1 performed poorly. Over all models, F-measure averaged 9.5% detection, with 16 to 23% detection in two-locus models, 9 to 15% detection in three-locus models, and less than 5% in four- and five-locus models. Specific detection was near 0% for all models. Allele frequency had no impact on detection. Geometric mean 1 performed slightly better than F-measure with 12% overall detection. Detection using this measure was sensitive to the allele frequencies specified in the genetic model. Detection in the 0.2 MAF models ranged between 24–34% in two-locus models, 14–24% in three-locus models, and 4–10% in four- and five-locus models. Detection in the 0.4 MAF models ranged between 15–19% in two-locus models, 10–13% in three-locus models, and 2–5% in four- and five-locus models. Specific detection using geometric mean 1 was near 0% for all models. Both F-measure and geometric mean 1 showed significantly worse detection and specific detection than classification error in nearly every case. Notably, these two measures outperformed all others for detection of two very difficult models (33 and 35), but in these cases the detection was very low and specific detection was no better than classification error.

The ROC-based measures Euclidean distance and geometric mean 2 also fail to outperform classification error overall, but perform well for some models. The average detection using Euclidean distance over all models was only 43.3%, but in two-locus models was near 100% except for the lowest heritability models (5 and 10). Across higher order models detection was generally scattered with no discernable trend, ranging from 2–77% in three-locus models, 3–46% in four-locus models, and 0–41% in five-locus models. Similarly, specific detection using Euclidean distance was between 90–99% for all two-locus models except the lowest heritability models (which were 80% for model 9 and 50% for model 10). Specific detection in higher order models was also scattered, but in general was higher for higher heritability models. Geometric mean 2 showed very similar trends for detection and specific detection to Euclidean distance. Detection was 85–100% for two-locus models, 13–70% for three-locus models, 6–20% in four-locus models and less than 7% in five-locus models. Specific detection was between 97 and 99% in all but the lowest heritability two-locus models. All others had less than 10% specific detection. Using Euclidean distance and geometric mean 2, only a few models show better detection than using classification error. The most notable of these is model 14 where geometric mean 2 improved over classification error by 41.67. Also, both Euclidean distance and geometric mean two showed significantly improved specific detection over classification error in all two-locus models except model 10.

The diagnostic testing measures Youden Index and Predictive Summary Index (PSI) perform relatively well. Overall, average detection was 64% using the Youden Index, and specific detection was 57%. Detection of two-locus models was 84–100%, 9.5–97% in three-locus models, 5–94% in four-locus models, and 0–95% in five-locus models. Specific detection was 90–92% in all two-locus models except the lowest heritability models which were 65–72%. Other models have higher specific detection in higher heritability models that decreases along with heritability. PSI also shows excellent overall average detection at 66.4% and specific detection at 59%. Detection was 84–100% in two-locus models, 9.5–97% in three-locus models, 7.4–94% in four-locus models, and 0–98% in five-locus models. The detection using PSI is lower in two-locus models than the Youden Index, but PSI shows slightly higher detection in a few three-, four- and five-locus models. Compared to classification error, PSI shows significantly increased detection in 23 of the 40 models. 8 models show no significant difference in detection, and for 9 models, classification error performs significantly better. Most of the improvement in detection is seen in higher order models, and notably, classification error detects as well or better then PSI for two-locus models. The Youden index shows better detection for 17 models. For 15 models, classification error is significantly better, and there is no significant difference for 8 models. Specific detection with PSI is improved over classification error in three-, four- and some five-locus models. Specific detection in two-locus models however is not consistently improved. Similar to PSI, the Youden index does not improve over classification error in two-locus models. Improvement in detection for the Youden index is in three- and four-locus models. Detection using the Youden index is not improved over classification error in five-locus models. Specific detection with the Youden index is significantly improved over classification error in all but five-locus models.

The chi-square and likelihood ratio statistical measures performed well. Using the chi-square, across all models average detection was 58.87% and average specific detection was 51.67%, with 78–100% detection for two-locus models, 11–82% detection for three-locus models, and 0–97% detection in four- and five-locus models with little discernable trend. Specific detection patterns also show no trend. Overall detection using the likelihood ratio is 65.36% and specific detection is 59.8%. In two-locus models, detection ranged from 84–100%, 16–96% in three-locus models, and 0–97% in four- and five-locus models. Using chi-square, detection of 15 models was significantly better than classification error, 10 models showed no significant difference in detection from classification error, and 15 models were detected significantly worse than using classification error. Specific detection was significantly better for 19 models using chi-square, was not significantly different from classification error for 4 models, and was significantly worse than error for 17 models. Most of the detection improvement from using the chi-square measure was seen in three- and four-locus models. Detection using likelihood ratio was significantly better or no different than error for all but 7 models, and specific detection was significantly improved for all models except five-locus models, where specific detection of 4 models was significantly worse using the likelihood ratio and was not statistically different for 2 of the models.

The information theoretic measures NMI and NMI-transpose (NMIT) both perform relatively well. Overall, detection using NMI was 65.36% across all models, and specific detection was 59.4%. With NMI, detection of two-locus models ranged from 84–100%, three-locus models from 16–96% and four- and five-locus models ranged from 0–98% detection. For specific detection the trends were very similar to those of the likelihood ratio, but specific detection was slightly higher in most cases. Using NMIT, overall detection was highest of all measures at 70.72%, with a specific detection of 59.8%. Per model detection rates were lower than with other measures, however, with two-locus model detection ranging from 45–98%, three-locus models from 23–98%, and four- and five-locus models ranging from 0–98%. Trends in specific detection were erratic, performing better than NMI in some three- and four-locus models, but much worse power for two-locus models. NMIT did demonstrate higher detection of higher order models. Detection using NMI is significantly better than classification error in 26 models, shows no difference in 8 models, and performs worse than classification error in 6 models. Specific detection is either significantly improved with NMI or shows no significant difference from classification error in all but 4 five-locus models. NMIT performs significantly worse than classification error for two-locus models, but shows significantly better detection for three- and four-locus models, with specific detection following the same trend.

In summary, Euclidean distance, F-measure, geometric mean 1, and geometric mean 2 perform significantly worse than classification error in both detection and specific detection for a majority of simulated models. Two measures, the chi-square and NMIT show improvement in detection and specific detection of some models, but are significantly worse than classification error for other models, with especially poor specific detection of two-locus models. The remaining four measures show either improved or equal detection and/or specific detection across a majority of models and work well for two-locus models. These measures, Youden index, predictive summary index, likelihood ratio, and normalized mutual information were evaluated for statistical power using permutation testing.

### Power

**Figure 5:**
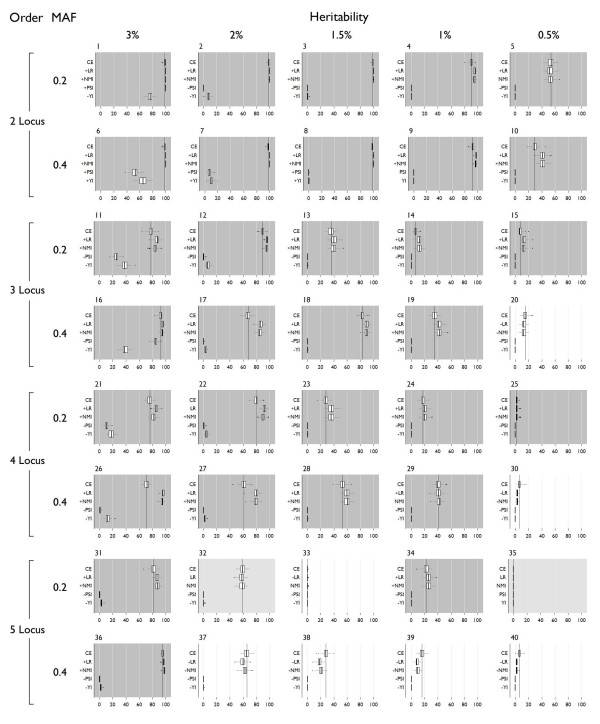
**Power box plots**. The dark vertical line indicates the mean detection using classification error. Dark shading indicates LR and NMI were significantly better, medium shading indicates no significant difference. Light shading indicates classification error was better than LR and NMI. "+" on the y axis indicates significantly different from classification error with a higher mean, "-" indicates significantly different from classification error with a lower mean. Significance was assigned by Wilcoxon rank-sum tests.

**Figure 6:**
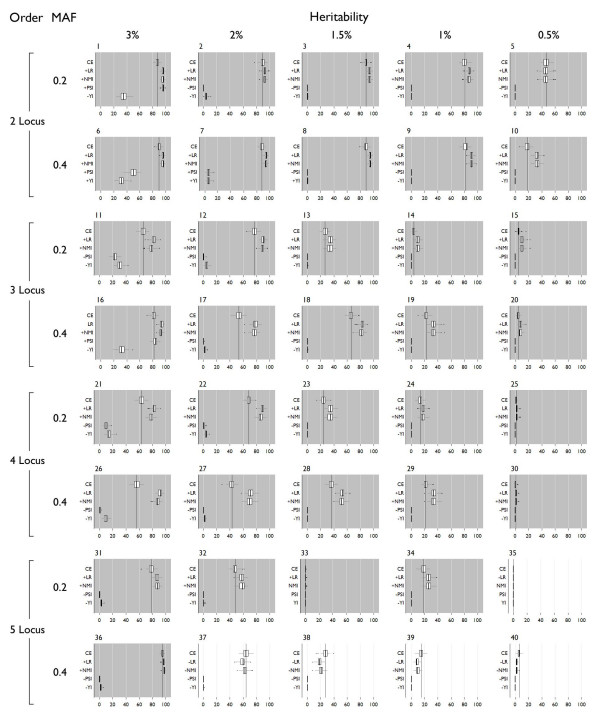
**Specific power box plots**. The dark vertical line indicates the mean detection using classification error. Dark shading indicates LR and NMI were significantly better, medium shading indicates no significant difference. Light shading indicates classification error was better than LR and NMI. "+" on the y axis indicates significantly different from classification error with a higher mean, "-" indicates significantly different from classification error with a lower mean. Significance was assigned by Wilcoxon rank-sum tests.

All power results are shown in Figure 5 and specific power is shown in Figure 6. "Power" is detection that is statistically significant (at α = 0.05), and "Specific power" is specific detection of the correct multi-locus model that is statistically significant (at α = 0.05). The process used to assign statistical significance to a result is dependent on the assumption that for these simulated data, the permutation distribution of a single dataset is equivalent to the permutation distribution of other datasets simulated using the same genetic model. After evaluating the variability of α = 0.05 critical values, this assumption holds well (see Additional file 1)

Predictive summary index (PSI) and the Youden index have power and specific power only in 3% and 2% heritability models – all other models show 0% power and specific power. PSI outperforms the Youden index for two-locus models for both power and specific power, but in higher order models, the Youden index performs slightly better. Neither of these measures shows improvement over classification error in the context of permutation testing.

Compared to classification error, the likelihood ratio shows significantly better power in 29 models, significantly worse power in 8 models, and no significant difference in 3 models. Of the 8 models where error was better, 6 were five-locus models, one was a 0.5% heritability three-locus model (model 20), and one was a 0.5% heritability two-locus model (model 5). Specific power of the likelihood ratio is significantly better than error for 33 models, significantly worse for 5 models, and not significantly different for 2 models. These five models are the 2-0.5% heritability five-locus models with 0.4 minor allele frequencies.

Using NMI, power is significantly better than error in 29 models, significantly worse for 7 models, and not significantly different in 4 models. Similar to the likelihood ratio, 5 of the 7 models are five-locus models, one was the same 0.5% heritability three-locus model (model 20), and one was a 0.5% heritability two-locus model (model 5). The same models showing significant differences in specific power from classification error for the likelihood ratio are also significantly different using NMI. Specific power is significantly better for 33 models, significantly worse for 4 models, and not significantly different for 3 models.

Comparing likelihood ratio and NMI, both show equal improvement over classification error for specific power. One model (32) showed significantly worse power compared to error when using the likelihood ratio, but power was not significantly different when using NMI. Power using likelihood ratio was significantly better than power using NMI for 2 three-locus models (11, 12), and 3 four-locus models (21, 22 and 26). Power using NMI was significantly better than power using likelihood ratio for 4 five-locus models (36–40). Specific power using likelihood ratio was significantly better for 9 models – 5 three-locus models (11, 12, 16, 17, and 18), and 2 four-locus models (26 and 27). NMI has significantly better specific power for 4 five-locus models (36–39).

## Discussion

This study is an exploration of fitness measures in the MDR algorithm. Here, we have evaluated ten alternative measures of fitness for MDR models and compared them to the traditional measure, classification error (or 1- classification accuracy). This work is highly motivated by the dispute over the use of classification error, as it is known to be an improper scoring rule. In addition, MDR has been released in a JAVA software package with a user-friendly Graphical User Interface version where many of these measures are currently available . We felt it was important to know which measures are robust for higher order interaction models.

The first series of simulations evaluated the traditional fitness measure for MDR, classification error, for a set of disease models. From these simulations, we observe some obvious trends in the two locus models as well as some irregular patterns in the higher order models. With the two-locus models, the detection and power of MDR using classification error decreases as the broad sense heritability of the model decreases.

Trends for higher order models are non-linear with respect to broad sense heritability. Most notably, five-locus models with 0.2 minor allele frequencies had a very erratic pattern, with the 1.5% and 0.5% heritability models (models 33 and 35) having near 0% power while the 1% heritability model has 22% power (model 34). These erratic patterns are likely due to one of two possible scenarios. One possibility is related to the way in which the 40 genetic models were created. The genetic algorithm-based procedure for creating multi-locus models with no marginal effects is a directed search through the space of all possible penetrance models. There may be millions of models which satisfy the heritability requirements, and we arrive at a random sample from that model space with each run of the procedure. Thus, there is some random variability between penetrance functions with similar heritabilites selected in this manner. This variability likely impacts the ability of MDR to detect the effect of that model, as MDR does not measure an effect based on the penetrance table directly, but rather on the derived two-way contingency table. A second possible scenario is that these penetrance models were generated with the assumption that there are no main effects of any single locus. However, in a higher-order models, the simulator does not test for all possible lower-order models. For example, if a three-locus model is being generated, there are no single locus effects for any of the three-loci; however, the software does not test for the presence of two-locus models embedded within the three loci. Thus, it is possible that the lower detection and power results are due to multiple models competing for detection. This was the case for model 14, for example, where models containing two of the three loci were detected in 70% of evaluations (data not shown). For this study, we consider the performance of classification error as a baseline, so while these effects are interesting to note, they are irrelevant for evaluating other classification measures. MDR using classification error has greater than 80% detection for 14 of the 40 multi-locus models, and greater than 80% power for 13 of the 40 models.

While high-order (three-, four-, or five-locus) models with no marginal effects have been speculated to exist, there is little confirmed experimental or statistical evidence to support their role in complex disease. It is encouraging to see however that MDR has appreciable power for many high-order models in a feasible sample size of 400 cases and 400 controls. If susceptibility to common disease truly does involve complex interactions among many variants, tools for detecting these interactions will be critical.

### Performance of Alternate Measures

The F-measure and geometric mean 1 both show similar detection and specific detection results across all models. These two measures combine precision and sensitivity in their calculation. In general these measures perform very poorly compared to classification error, indicating that information about the specificity of an MDR model improves the performance of MDR. These measures are focused on classifying cases correctly. An MDR model that classifies cases best is not necessarily based on the most associated genetic factors. The model would not discern the difference between genetic factors strongly associated to both cases and controls and only those associated to cases.

Geometric mean 2 and Euclidean distance are similar in that they are geometric functions of sensitivity and specificity, functioning similarly to a receiver-operator characteristic analysis. These measures perform commensurately with classification error for two-locus models, but do not perform especially well for higher order interactions. Euclidean distance in general performs better than geometric mean 2 for both detection and specific detection. The one notable exception to this trend is geometric mean 2, which shows very good specific detection in two-locus models with 0.2 MAF, out-performing all other measures (Figure 2, 1, 2, 3, 4, 5).

The diagnostic measures Youden index and PSI perform well in detection and specific detection. The Youden index seems to show better detection than PSI in two-locus models and high heritability three-locus models. PSI however shows better specific detection over all models. Both measures out-perform classification error in both detection and specific detection, but those measures do not show improved power or specific power over classification error. One explanation could be that the empirical distribution of these statistics was not as stable (particularly in the tail region), so the standardized distribution for each model failed to properly assign statistical significance. While the empirical distribution of randomly selected datasets did not differ significantly from the standardized distribution, some subtle variability in the tail regions was noted. Another possibility is that these measures are more susceptible to noise in the data. The power and specific power are stronger for 3% heritability models than for models with lower heritability. While these measures still have utility for detection, their usefulness when assigning statistical significance is questionable.

The chi-square shows good detection and specific detection in the four-locus models and a few five-locus models, but fails to out-perform CE in most other cases. One reason why the chi-square does not perform well in this setting is that theoretically, the chi-square is not a satisfactory measure of association, and may not rank MDR results to produce optimal detection [37]. The chi-square tests deviation from independence, but does not necessarily quantify the strength of an association. This is an important consideration for using the chi-square test in other studies, particularly whole-genome association studies, as the chi-square may not necessarily rank signals by strength of association

The measures that demonstrate the most consistent improved performance are the likelihood ratio and NMI. While the improvement is not dramatic, these measures show equal or better detection and power across nearly all models. The more dramatic improvement is in specific detection and specific power, where the genetic model detected by MDR is the exact model that was simulated. This is an interesting result because this means that MDR using NMI or LR is less susceptible to over-fitting (including more variables in the model than necessary).

Both the likelihood ratio and NMI measures are based on entropy, which is loosely analogous to variance in the two-way table, and both measures show very similar trends for detection and power. These two measures are ultimately related, as the numerator of NMI is a transformation of the LR [37].

(6) $$H(x)-H(x|y)=\frac{LR}{2(\ln2)p••}$$

The likelihood ratio test is a well-established statistical test to examine a sample's deviation from a null hypothesis. The statistic itself however does not have an intuitive interpretation, and often is transformed to achieve a valid measure of association [37].

NMI of the contingency table treats the "true outcome" and the "model prediction" as a pair of two-state random variables. NMI quantifies the amount of uncertainty (or entropy) about the state of the "truth variable" removed by the "model prediction variable". NMI has a nice interpretability as the amount by which the model reduces our uncertainty about the true state. While NMIT performs well for three-locus models in particular and did outperform classification error in many cases, its poor detection and specific detection of two-locus models makes it an unattractive measure. In addition, NMIT's interpretation makes less sense as the amount the true variable reduces our uncertainty about the model.

NMI includes details of the contingency table not accounted for by the other measures of model predictability. For example, the numerator of NMI takes into account the power to correctly predict both the cases and controls: A/(A+B) and D(C+D), respectively. Explicitly, we can rewrite part of the NMI numerator terms as

(7) $$A\ln\left(\frac{A}{A+B}\right)+D\ln\left(\frac{D}{C+D}\right)$$

which is closely related to Predictive Summary Index (PSI). In addition, we can rewrite part of the NMI denominator as

(8) $$A\ln\left(\frac{A}{A+C}\right)+D\ln\left(\frac{D}{B+D}\right)$$

which bears strong resemblance to the Youden Index in its attempt to balance the model sensitivity and specificity: A/(A+C) and D/(B+D), respectively. The detailed form of the NMI measure likely leads to its observed ability to distinguish between closely similar high quality models; and hence, NMI's improved ability to uniquely determine the relevant variables (*i.e*., its ability to achieve higher specific power). Also, NMI preferentially selects models that classify either cases or controls perfectly (or nearly perfectly). These models are more "stable", or less variable, and are thus to be preferred by the measure over models where both cases and controls are misclassified equally. The dependence of NMI on the contingency table is quite intricate and warrants further investigation to understand its strengths and limitations more fully.

While the power improvement using NMI or the likelihood ratio in most cases is not dramatic, these measures are superior to classification error. Using these measures, there is higher detection and power of high-order interactions and better specific power overall, so an analyst can be more confident that an MDR model does not contain spurious variables. Also, classification error assumes the distribution of the two classes to be equal. This shortcoming was recently addressed by Velez et al. [23] who used an average of sensitivity and specificity to compute a balanced classification error (or balanced accuracy) for MDR model evaluation, and demonstrated its power to detect gene-gene interactions in cases of class imbalance. Both NMI and LR also take into account the sensitivity and specificity of an MDR model, and likewise should not be susceptible to class imbalance. One clear advantage of classification error is its interpretability. Of the two improved measures, NMI perhaps has the easiest interpretation. Its value ranges from 0 to 1, with 0 meaning the genotype and status are independent and 1 meaning the genotype completely determines the status. Also, as NMI provides a direct information theoretical measure of association, it may be preferred over the likelihood ratio test statistic, which measures deviation from the null hypothesis of independence rather than directly quantifying the degree of association. For these reasons, we recommend that NMI be used in lieu of classification error for MDR analyses. For clarity of interpretation, we recommend showing the two-way contingency table along with reporting the NMI of an MDR result.

Classification error (or classification accuracy) is a widely used measure of performance in many areas of research. The results of this study are specific to classification using the MDR procedure, but this work does provide additional empirical evidence to support general theoretical arguments against the use of classification error [26-29].

### Limitations

There are some limitations of this work. First, this study was conducted using simulated data, so confidence in the results relies on the quality of the simulated data. All disease loci in a genetic model were simulated with the same minor allele frequency, either 0.2 or 0.4, which is a simplification. The biological relevance of the penetrance functions simulated could be questioned, though we use functions with very small marginal effects as a "worst-case scenario" and expect the results of this study to generalize to situations where marginal effects are detectable. It is troubling that the trends observed in power and detection do not always follow the expected trends given the broad-sense heritabilities simulated. However, as this study is focused on the relative performance of classification measures, this point is not critical. Permutation testing for all 400,000 models evaluated in this study was not computationally feasible, and a single permutation distribution for each of the 40 models was generated for the 5 measures evaluated for statistical power. These permutation distributions did not appear to vary significantly by QQ-plot from a randomly seeded dataset using the same model. Assigning significance using a permutation distribution uses the tails of the distribution, however, and some variability in the tails was observed in the QQ-plots. But the power results do closely follow detection results, and it seems unlikely that all 40 of the permutation distributions used would consistently over estimate or under estimate the tail values. The simulated data included only ten loci total. While small-scale studies are still performed, low-cost genotyping solutions have dramatically increased the number of polymorphisms examined in a typical study. While the computation time required to perform this study prohibited using a large number of SNPs for this evaluation, we expect that the relative performance of these fitness measures would extend to datasets with larger numbers of SNPs. Absolute detection and power, however, are influenced by a variety of factors including the number of SNPs in the dataset.

## Conclusion

Over a variety of simulated genetic models, normalized mutual information (NMI) and the likelihood ratio demonstrate stellar performance as measures of Multifactor Dimensionality Reduction model fitness; an improvement in comparison to classification error. The ability of MDR to specifically detect only the simulated disease loci was significantly higher using these two measures for nearly all genetic models. These measures also show improved statistical power by permutation testing. Of these measures, NMI is perhaps easier to interpret, as it is the amount by which the model reduces our uncertainty about the true disease state. NMI properly treats imbalanced data and provides superior performance over classification error. Therefore, we recommend using NMI as an alternative to classification error for MDR analyses.

## Methods

### MDR algorithm

The MDR procedure is outlined in Figure 1. In step 1 of the process, the data are divided into a training set (4/5 of the data) and a testing set (1/5 of the data). Next, we select the order of interaction *n *to assess, where a second-order model consists of two genetic factors (step 2). In step 3, variable combinations are constructed by selecting a set of *n *genetic and/or environmental factors from the set of all possible factors N. Each individual in the training set is grouped according to its state at each of the *n *factors (i.e. for 2 loci with 3 possible genotypes, there are 9 possible combinations; for 3 loci, 27 etc.) Then, each genotype combination is classified as high- or low-risk depending on the ratio of cases to controls with that genotype combination (step 4). A threshold T is defined as the ratio of cases to controls in the data, so T = 1 for balanced data. If the ratio is < T (there are less cases than controls) the genotype combination is classified as low risk. If the ratio is ≥ T, the genotype combination is classified as high-risk.

The collection of high- and low-risk multi-locus genotype groups defines the MDR model for that combination of factors (step 5). When compared to the true affection status, the score or fitness of a model can be represented as a two-way contingency table in which the true and classified status are treated as variables(step 6). Typically, this two-way table is mathematically transformed to a single value. Classification error, for instance, is the sum of the diagonal divided by the total N in the training set (unaffected high-risk individuals + affected low-risk individuals/total N) (figure 2B). Each variable combination produced in step 3 is evaluated in this fashion.

The combination of variables with the lowest classification error is selected (step 7), and a prediction error is calculated using the testing set by comparing the sum of the unaffected individuals predicted to be high-risk and the affected individuals predicted to be low risk divided by the total N in the testing set (step 8). Variable combinations are generated and evaluated in this manner for each order evaluation specified in step 2. The lowest classification error model and its associated prediction error are recorded for each order evaluation that is performed.

The entire procedure is performed 5 times, using each 1/5th as a testing set and each 4/5th as a training set. This cross-validation (CV) procedure produces 5 sets of results, one for each CV interval. A model is selected for each order, and the average prediction error and cross-validation consistency (CVC) are computed for each (step 9). The CVC is the number of cross-validation intervals producing the model.

From this set of models (one for each order evaluation), the model with the highest cross-validation consistency and the lowest average prediction error is selected as the best overall model (step 10). In the event that two models have equal measures, the model with the fewest number of loci is chosen, as it is the most parsimonious.

The statistical significance of this best overall model is established by Monte Carlo permutation testing (step 11). K datasets are generated by randomly reassigning the affection status of individuals in the dataset (K is typically > = 1000). The entire MDR process is then repeated for each of these K replicates to sample the empirical distribution of average prediction error statistics. This distribution is used to assign significance to the average prediction error of the best overall model from the analysis of the original dataset. The MDR process establishes the fitness of a given model based on classification error and prediction error.

### Simulations

To properly evaluate the performance of various fitness measures within the MDR process, data with known patterns of association were required. To accomplish this, we simulated 40 penetrance functions using a genetic algorithm approach (see Additional file 1) [38]. Penetrance functions range in the number of disease model loci (2, 3, 4, 5 loci) and in the proportion of trait variance explained by genotypes using "broad sense" heritability (3%, 2%, 1.5%, 1%, 0.5% heritability) described by Culverhouse et al. [24]. Alzheimer's disease, an example of a common complex disease, has an estimated heritability between 58% and 79% [39], with polygenetic inheritance. For this study, our simulated genetic models assume that a small set of genetic loci (fewer than five) interact to account for a small proportion of the overall heritability. Penetrance functions were generated using two minor allele frequencies, 0.2 and 0.4. Using the genomeSIM software [40], these penetrance functions were used to simulate datasets consisting of 400 cases and 400 controls with a total of ten independent loci. Non-disease loci allele frequencies varied randomly from 0.05 to 0.5. Disease loci allele frequencies were set to match the frequencies specified by the penetrance function. We chose to simulate a small set of SNPs for each dataset because the goal of this study is to evaluate the relative detection and power of various fitness measures. This required repeating many replicates, and the computation time required to perform this experiment prohibited using a large number of SNPs. We expect that these results would extend to datasets with larger numbers of SNPs. We simulated 100 datasets for each genetic model for a total of 4,000 datasets per experiment. We conducted 100 replicate experiments, for a total of 400,000 datasets.

### Analysis

We modified a C++ version of the MDR software to accept multiple measures of fitness; classification error, geometric mean 1, F-measure, geometric mean 2, Euclidean distance, Youden index, predictive summary index, chi-square test of association, likelihood ratio test, normalized mutual information, and normalized mutual information transpose. With each fitness measure evaluation, the selected measure was used instead of classification error to rank models during the training phase, and instead of prediction error to rank models during the testing phase. All other aspects of the algorithm are unchanged. Run-time for examining 2 through 5 locus models with permutation testing using the MDR algorithm was less than 1 hour on a typical workstation computer (2.0 GHz Opteron), regardless of fitness measure. Run-time scales exponentially with the number of variables in the dataset.

To evaluate these measures, several quantities were defined. "Detection" is the ability of the method to correctly identify all disease loci, but additional non-disease loci may be included in the model also. "Specific detection" is the ability of the method to correctly identify all the disease loci and no additional loci – this could also be phrased as the ability to detect the correct multi-locus model. These values are empirically defined as a proportion of MDR evaluations out of 100 datasets where the condition holds. We conducted this analysis in 100 replicate experiments to provide more accurate estimates of detection values (for a total of 400,000 results). For selected measures, we used permutation testing to assign statistical significance to each MDR result produced in the study. "Power" is detection that is statistically significant (at α = 0.05), and "Specific power" is specific detection of the correct disease model that is statistically significant (at α = 0.05), Wilcoxon Rank-sum tests were used to formally compare detection and power results for two measures. Statistical significance was achieved with a Wilcoxon Rank-sum p-value < 0.05. All statistical analyses were performed using STATA 9.1.

Permutation testing, which is used to establish statistical significance of an MDR result, is a computationally intensive process. To circumvent the computational burden of performing permutation tests for each of the 400,000 data set evaluations, we produced one standard empirical distribution (K = 10,000) for each of the 40 genetic models. Thus, the result from each of the 10,000 dataset evaluations was assigned statistical significance from one standard empirical distribution generated based on the genetic model used to simulate that data. To evaluate this assumption, we generated the permutation distributions of 10 randomly generated datasets under each genetic model, for each fitness measure. We then estimated the distribution of α = 0.05 critical values collected from these 10 permutation distributions, and recomputed power and specific power using the 5^th ^and 95^th ^percentiles of the distribution (see Additional file 1). Using these values does not change the relative rankings of power or specific power results.

## Authors' contributions

WSB designed the study, performed statistical analysis, and drafted the manuscript. TLE performed statistical analysis. SMD provided software and scripting support. BAM performed theoretical statistical analysis and helped draft the manuscript. MDR conceived of the study, participated in its design and coordination, and helped draft the manuscript. All authors read and approved the final manuscript.

## Supplementary Material

Additional file 1 Statistical details of detection, power, and genetic models. The figures and data tables provided show the multi-locus penetrance tables used for each genetic model, the statistical details of the detection and power results, and confidence bounds for the power results. Click here for file
