# Supplementary material for: Alternative contingency table measures improve the power and detection of multifactor dimensionality reduction
Source: BMC Bioinformatics. 2008 May 16;9:238. doi: 10.1186/1471-2105-9-238 (PMC2412877; doi:10.1186/1471-2105-9-238)
Supplement: Additional file 1 — Statistical details of detection, power, and genetic models. The figures and data tables provided show the multi-locus penetrance tables used for each genetic model, the statistical details of the detection and power results, and confidence bounds for the power results. [file 1471-2105-9-238-S1.doc]

Supplemental Figure 1. Power Results Computed using 5^th^ and 95^th^ Percentile Critical Values. Using 10 randomly selected permutation distributions under each model and for each measure, 5^th^ and 95^th^ percentile estimates of critical values were generated and used to compute power.


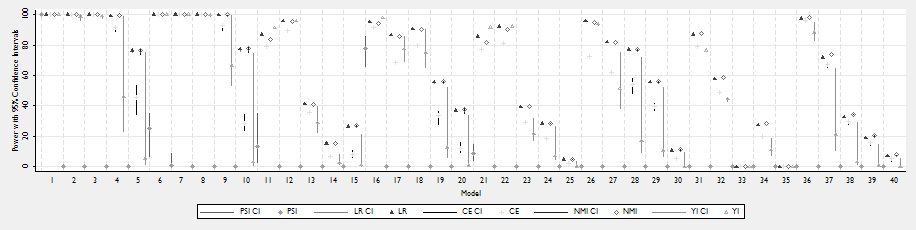


Supplemental Figure 2. Specific Power Results Computed using 5^th^ and 95^th^ Percentile Critical Values. Using 10 randomly selected permutation distributions under each model and for each measure, 5^th^ and 95^th^ percentile estimates of critical values were generated and used to compute specific power.


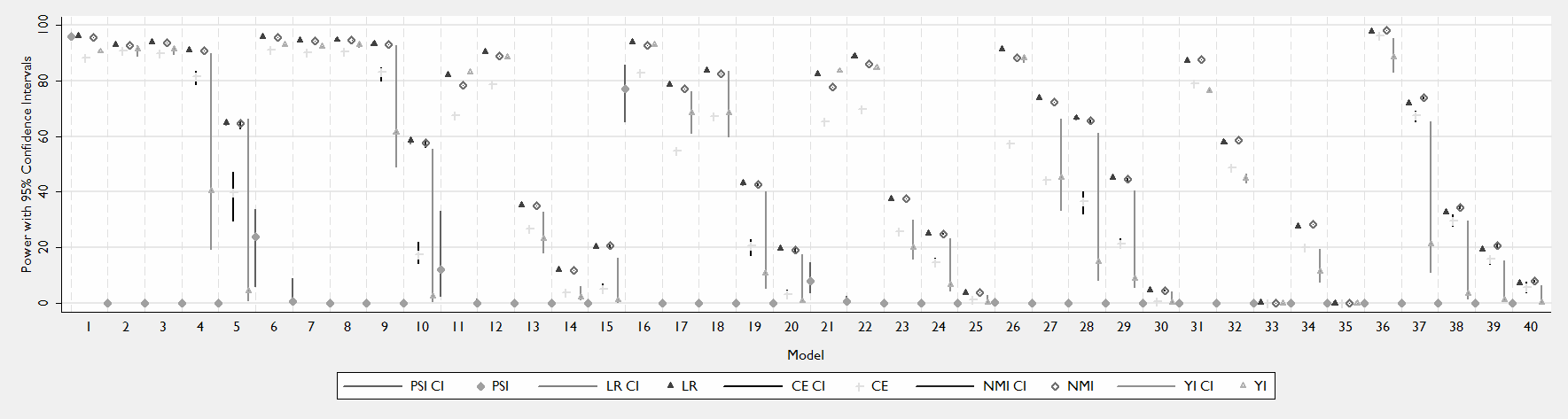


Supplemental Table 1. Detection Results

| **Detection** | | | | | | | | | | | | | | | | | | | | | | |
| --- | --- | --- | --- | --- | --- | --- | --- | --- | --- | --- | --- | --- | --- | --- | --- | --- | --- | --- | --- | --- | --- | --- |
|  | **CE** | | **PSI** | | **LR** | | **G1** | | **G2** | | **F** | | **NMIT** | | **X2** | | **EU** | | **NMI** | | **YI** | |
| Model | Mean | Std Dev | Mean | Std Dev | Mean | Std Dev | Mean | Std Dev | Mean | Std Dev | Mean | Std Dev | Mean | Std Dev | Mean | Std Dev | Mean | Std Dev | Mean | Std Dev | Mean | Std Dev |
| 1 | 100.00 | 0.00 | 100.00 | 0.00 | 100.00 | 0.00 | 24.70 | 4.47 | 100.00 | 0.00 | 20.56 | 3.90 | 98.52 | 1.14 | 100.00 | 0.00 | 100.00 | 0.00 | 100.00 | 0.00 | 100.00 | 0.00 |
| 2 | 99.99 | 0.10 | 99.93 | 0.26 | 100.00 | 0.00 | 28.75 | 4.66 | 99.89 | 0.35 | 21.23 | 4.09 | 98.75 | 1.06 | 99.99 | 0.10 | 99.92 | 0.27 | 100.00 | 0.00 | 100.00 | 0.00 |
| 3 | 100.00 | 0.00 | 99.92 | 0.27 | 100.00 | 0.00 | 26.28 | 4.15 | 99.69 | 0.54 | 22.63 | 4.33 | 99.05 | 0.93 | 100.00 | 0.00 | 99.19 | 0.84 | 100.00 | 0.00 | 100.00 | 0.00 |
| 4 | 98.91 | 1.07 | 98.52 | 1.10 | 99.62 | 0.56 | 29.75 | 5.01 | 98.87 | 1.04 | 22.30 | 4.31 | 88.59 | 3.40 | 99.08 | 1.03 | 98.65 | 1.23 | 99.65 | 0.54 | 99.52 | 0.80 |
| 5 | 79.95 | 4.26 | 75.04 | 3.89 | 83.63 | 3.66 | 34.39 | 4.85 | 87.81 | 3.53 | 20.28 | 3.82 | 45.98 | 5.40 | 78.40 | 4.01 | 83.39 | 3.85 | 84.56 | 3.57 | 83.66 | 4.01 |
| 6 | 100.00 | 0.00 | 99.99 | 0.10 | 100.00 | 0.00 | 16.92 | 3.68 | 100.00 | 0.00 | 16.72 | 4.23 | 99.98 | 0.14 | 100.00 | 0.00 | 100.00 | 0.00 | 100.00 | 0.00 | 100.00 | 0.00 |
| 7 | 100.00 | 0.00 | 100.00 | 0.00 | 100.00 | 0.00 | 15.89 | 3.80 | 99.99 | 0.10 | 17.64 | 4.00 | 99.86 | 0.38 | 100.00 | 0.00 | 99.91 | 0.35 | 100.00 | 0.00 | 100.00 | 0.00 |
| 8 | 100.00 | 0.00 | 99.97 | 0.17 | 100.00 | 0.00 | 16.63 | 3.64 | 99.99 | 0.10 | 16.42 | 3.82 | 99.68 | 0.58 | 100.00 | 0.00 | 99.95 | 0.22 | 100.00 | 0.00 | 100.00 | 0.00 |
| 9 | 99.95 | 0.22 | 99.64 | 0.61 | 99.97 | 0.17 | 16.99 | 4.12 | 99.52 | 0.70 | 16.89 | 3.37 | 95.09 | 2.14 | 99.63 | 0.58 | 99.26 | 0.81 | 99.97 | 0.17 | 99.96 | 0.20 |
| 10 | 92.69 | 2.78 | 88.53 | 3.27 | 91.91 | 2.90 | 19.89 | 3.95 | 84.99 | 3.62 | 18.33 | 3.84 | 62.93 | 4.50 | 78.07 | 3.95 | 86.25 | 2.92 | 92.52 | 2.83 | 92.05 | 2.68 |
| 11 | 78.84 | 3.81 | 84.26 | 3.48 | 86.89 | 3.56 | 14.51 | 3.60 | 48.22 | 5.45 | 12.06 | 3.31 | 98.54 | 1.33 | 54.61 | 5.17 | 57.37 | 5.56 | 83.74 | 3.82 | 91.41 | 2.71 |
| 12 | 89.26 | 3.05 | 96.24 | 1.89 | 95.94 | 1.97 | 17.75 | 4.03 | 38.10 | 4.56 | 13.81 | 3.60 | 99.69 | 0.61 | 81.77 | 3.76 | 58.16 | 5.05 | 95.16 | 2.11 | 95.88 | 2.02 |
| 13 | 38.03 | 4.80 | 39.04 | 4.61 | 41.44 | 5.36 | 19.46 | 4.10 | 19.78 | 3.82 | 14.13 | 3.60 | 60.74 | 5.25 | 11.86 | 3.29 | 22.89 | 4.07 | 41.29 | 5.18 | 40.53 | 4.90 |
| 14 | 9.58 | 3.05 | 24.94 | 4.69 | 15.82 | 3.81 | 22.95 | 4.29 | 51.25 | 4.54 | 11.71 | 3.53 | 36.49 | 4.75 | 12.27 | 3.24 | 1.73 | 1.21 | 15.84 | 3.76 | 9.44 | 3.14 |
| 15 | 25.51 | 4.49 | 34.36 | 4.58 | 31.32 | 4.46 | 24.21 | 4.43 | 26.16 | 4.59 | 14.62 | 3.39 | 23.10 | 3.64 | 26.05 | 4.64 | 12.36 | 3.57 | 31.90 | 4.61 | 28.15 | 5.18 |
| 16 | 91.83 | 2.81 | 91.89 | 2.31 | 95.25 | 2.27 | 13.12 | 3.39 | 52.63 | 4.84 | 9.51 | 2.70 | 98.43 | 1.23 | 47.39 | 4.35 | 77.14 | 4.04 | 94.34 | 2.39 | 97.69 | 1.36 |
| 17 | 69.71 | 4.64 | 86.63 | 3.18 | 86.63 | 3.66 | 11.68 | 3.11 | 13.34 | 3.53 | 12.11 | 2.97 | 97.28 | 1.41 | 34.40 | 4.49 | 32.90 | 4.21 | 85.67 | 3.61 | 86.11 | 3.66 |
| 18 | 81.59 | 3.45 | 89.60 | 3.20 | 90.42 | 2.95 | 10.39 | 2.84 | 68.95 | 4.51 | 10.74 | 2.99 | 95.89 | 1.89 | 42.50 | 4.87 | 49.55 | 5.02 | 90.00 | 3.10 | 91.13 | 2.39 |
| 19 | 52.75 | 5.54 | 65.05 | 4.29 | 57.79 | 5.02 | 12.49 | 3.30 | 17.11 | 4.49 | 12.44 | 3.34 | 71.41 | 4.48 | 32.12 | 4.60 | 33.15 | 5.40 | 58.11 | 4.89 | 55.80 | 5.17 |
| 20 | 55.53 | 4.95 | 54.53 | 5.34 | 47.54 | 5.41 | 11.70 | 3.12 | 38.72 | 4.23 | 12.26 | 3.14 | 36.56 | 4.43 | 25.69 | 4.84 | 44.54 | 5.27 | 48.42 | 5.32 | 50.46 | 5.34 |
| 21 | 76.75 | 4.19 | 78.30 | 4.00 | 85.73 | 3.43 | 6.20 | 2.43 | 12.60 | 3.18 | 3.74 | 1.85 | 88.22 | 3.12 | 89.70 | 2.68 | 5.36 | 2.44 | 81.58 | 3.85 | 91.50 | 2.77 |
| 22 | 80.81 | 3.85 | 79.52 | 4.06 | 92.13 | 2.73 | 8.14 | 2.73 | 10.58 | 3.17 | 4.96 | 2.49 | 85.13 | 3.69 | 93.56 | 2.55 | 13.22 | 3.64 | 89.95 | 2.98 | 92.21 | 2.80 |
| 23 | 32.17 | 4.36 | 45.70 | 5.06 | 39.64 | 5.08 | 10.30 | 2.97 | 6.14 | 2.51 | 4.90 | 2.22 | 54.52 | 4.89 | 39.65 | 5.01 | 10.99 | 3.16 | 39.91 | 5.16 | 32.87 | 4.80 |
| 24 | 27.69 | 4.76 | 29.31 | 4.33 | 29.30 | 4.73 | 10.48 | 2.74 | 10.41 | 3.34 | 4.70 | 2.03 | 26.82 | 4.39 | 31.34 | 4.84 | 14.31 | 3.88 | 29.63 | 4.67 | 28.54 | 4.52 |
| 25 | 5.08 | 2.40 | 7.35 | 2.52 | 5.59 | 2.39 | 9.40 | 2.81 | 10.59 | 3.44 | 5.30 | 2.32 | 6.11 | 2.09 | 7.51 | 2.82 | 2.83 | 1.58 | 5.79 | 2.36 | 4.70 | 2.32 |
| 26 | 72.53 | 4.33 | 93.94 | 2.55 | 96.01 | 2.05 | 4.51 | 2.09 | 8.04 | 3.03 | 4.53 | 2.15 | 94.41 | 2.44 | 96.14 | 1.88 | 44.97 | 4.01 | 94.53 | 2.46 | 94.54 | 2.16 |
| 27 | 65.39 | 5.01 | 85.37 | 3.43 | 82.13 | 4.02 | 5.07 | 1.87 | 5.94 | 2.13 | 4.69 | 2.08 | 87.21 | 3.43 | 82.17 | 3.83 | 26.76 | 4.45 | 81.94 | 3.95 | 76.28 | 4.53 |
| 28 | 74.18 | 4.37 | 83.78 | 3.47 | 79.22 | 3.91 | 4.89 | 2.16 | 13.36 | 3.25 | 4.92 | 2.15 | 78.66 | 3.99 | 79.01 | 4.01 | 46.62 | 4.88 | 79.57 | 3.97 | 76.47 | 4.02 |
| 29 | 56.81 | 4.38 | 63.62 | 4.47 | 57.86 | 4.44 | 5.09 | 2.25 | 20.94 | 4.25 | 4.43 | 2.07 | 61.68 | 3.97 | 60.65 | 4.19 | 32.53 | 5.08 | 58.46 | 4.38 | 55.95 | 4.73 |
| 30 | 24.86 | 4.14 | 19.57 | 3.90 | 14.84 | 3.69 | 5.30 | 2.07 | 14.04 | 3.35 | 4.79 | 2.23 | 12.03 | 3.26 | 16.21 | 3.91 | 19.50 | 3.93 | 15.66 | 3.87 | 17.67 | 3.83 |
| 31 | 78.91 | 4.58 | 87.38 | 3.48 | 86.99 | 3.42 | 4.21 | 2.01 | 3.92 | 1.69 | 1.30 | 1.13 | 89.39 | 3.32 | 87.39 | 3.56 | 10.06 | 3.09 | 87.68 | 3.36 | 76.72 | 4.86 |
| 32 | 48.80 | 5.18 | 57.94 | 4.59 | 57.73 | 4.76 | 3.71 | 1.93 | 3.42 | 1.66 | 1.37 | 1.20 | 59.07 | 4.47 | 72.77 | 4.39 | 6.24 | 2.55 | 58.55 | 4.82 | 46.42 | 4.93 |
| 33 | 0.28 | 0.57 | 0.25 | 0.50 | 0.14 | 0.38 | 4.19 | 1.74 | 1.68 | 1.32 | 2.06 | 1.69 | 0.10 | 0.30 | 0.32 | 0.55 | 0.25 | 0.52 | 0.16 | 0.39 | 0.15 | 0.48 |
| 34 | 21.79 | 4.82 | 32.07 | 5.01 | 27.68 | 4.79 | 4.40 | 1.86 | 3.15 | 1.59 | 1.68 | 1.44 | 34.57 | 5.02 | 34.61 | 4.55 | 4.57 | 2.20 | 28.27 | 4.82 | 19.53 | 4.35 |
| 35 | 0.07 | 0.29 | 0.04 | 0.24 | 0.04 | 0.20 | 4.13 | 1.78 | 1.57 | 1.30 | 2.19 | 1.35 | 0.01 | 0.10 | 0.13 | 0.34 | 0.06 | 0.24 | 0.04 | 0.20 | 0.05 | 0.26 |
| 36 | 96.07 | 1.76 | 98.42 | 1.33 | 97.54 | 1.62 | 2.15 | 1.35 | 6.64 | 2.76 | 1.26 | 1.18 | 98.53 | 1.31 | 97.57 | 1.55 | 41.85 | 5.03 | 97.95 | 1.51 | 95.29 | 1.92 |
| 37 | 72.24 | 4.37 | 79.55 | 3.49 | 72.87 | 4.25 | 1.89 | 1.31 | 5.40 | 2.46 | 1.65 | 1.17 | 75.56 | 3.62 | 75.17 | 4.20 | 28.08 | 4.73 | 74.66 | 4.12 | 66.91 | 4.29 |
| 38 | 40.07 | 4.68 | 43.28 | 4.94 | 34.94 | 4.44 | 1.97 | 1.54 | 5.19 | 1.96 | 1.46 | 1.12 | 34.69 | 4.78 | 35.26 | 4.75 | 19.22 | 3.72 | 36.60 | 4.45 | 33.69 | 4.74 |
| 39 | 25.77 | 4.62 | 29.16 | 4.77 | 21.19 | 4.04 | 2.22 | 1.32 | 4.41 | 1.85 | 1.49 | 1.14 | 21.94 | 4.10 | 21.95 | 4.28 | 2.13 | 4.24 | 22.91 | 4.24 | 18.88 | 3.46 |
| 40 | 15.56 | 3.76 | 13.05 | 3.83 | 8.85 | 3.01 | 2.10 | 1.25 | 5.00 | 2.10 | 1.61 | 1.10 | 7.68 | 2.87 | 9.98 | 3.21 | 0.00 | 0.00 | 9.53 | 3.26 | 10.38 | 2.90 |

Supplemental Table 2. Specific Detection Results

| **Specific Detection** | | | | | | | | | | | | | | | | | | | | | | |
| --- | --- | --- | --- | --- | --- | --- | --- | --- | --- | --- | --- | --- | --- | --- | --- | --- | --- | --- | --- | --- | --- | --- |
|  | **CE** | | **PSI** | | **LR** | | **G1** | | **G2** | | **F** | | **NMIT** | | **X2** | | **EU** | | **NMI** | | **YI** | |
| Model | Mean | Std Dev | Mean | Std Dev | Mean | Std Dev | Mean | Std Dev | Mean | Std Dev | Mean | Std Dev | Mean | Std Dev | Mean | Std Dev | Mean | Std Dev | Mean | Std Dev | Mean | Std Dev |
| 1 | 88.20 | 2.87 | 95.69 | 1.84 | 96.06 | 1.95 | 0.26 | 0.44 | 99.74 | 0.48 | 0.00 | 0.00 | 80.91 | 4.15 | 93.37 | 2.51 | 99.30 | 0.83 | 95.65 | 1.98 | 90.48 | 2.86 |
| 2 | 90.66 | 3.13 | 90.98 | 2.86 | 92.87 | 2.32 | 0.01 | 0.10 | 98.81 | 1.04 | 0.00 | 0.00 | 68.46 | 4.65 | 78.43 | 4.44 | 98.05 | 1.39 | 92.56 | 2.28 | 92.59 | 2.59 |
| 3 | 89.82 | 2.74 | 93.52 | 2.45 | 93.93 | 2.54 | 0.04 | 0.20 | 98.50 | 1.28 | 0.00 | 0.00 | 76.05 | 4.38 | 76.39 | 4.71 | 94.92 | 2.09 | 93.66 | 2.65 | 91.82 | 2.83 |
| 4 | 88.06 | 3.22 | 79.73 | 3.60 | 91.16 | 2.66 | 0.37 | 0.56 | 97.24 | 1.72 | 0.04 | 0.20 | 29.57 | 4.42 | 87.71 | 3.61 | 95.68 | 2.16 | 90.98 | 2.66 | 90.08 | 2.88 |
| 5 | 68.36 | 4.77 | 47.31 | 4.96 | 70.77 | 4.39 | 1.12 | 1.15 | 81.65 | 4.40 | 0.17 | 0.43 | 9.63 | 2.99 | 66.04 | 4.66 | 78.46 | 4.07 | 71.24 | 4.32 | 71.91 | 4.77 |
| 6 | 91.21 | 2.18 | 96.67 | 1.68 | 95.60 | 2.16 | 0.04 | 0.20 | 98.17 | 1.53 | 0.00 | 0.00 | 95.21 | 2.11 | 78.45 | 4.20 | 98.61 | 1.12 | 95.42 | 2.25 | 92.99 | 2.12 |
| 7 | 89.95 | 3.23 | 96.28 | 1.76 | 94.42 | 2.02 | 0.11 | 0.31 | 98.00 | 1.46 | 0.00 | 0.00 | 92.49 | 2.66 | 72.74 | 4.22 | 97.44 | 1.69 | 94.31 | 2.07 | 92.30 | 2.66 |
| 8 | 90.54 | 3.19 | 95.49 | 2.19 | 94.61 | 2.09 | 0.05 | 0.22 | 97.98 | 1.33 | 0.00 | 0.00 | 87.06 | 3.60 | 71.90 | 4.69 | 97.39 | 1.72 | 94.42 | 2.09 | 93.26 | 2.65 |
| 9 | 89.58 | 3.16 | 89.23 | 3.06 | 93.34 | 2.74 | 0.05 | 0.22 | 95.09 | 2.39 | 0.07 | 0.26 | 54.40 | 4.86 | 85.38 | 3.53 | 90.48 | 3.19 | 93.15 | 2.82 | 92.71 | 2.61 |
| 10 | 62.04 | 5.01 | 52.15 | 4.78 | 68.55 | 5.07 | 1.21 | 1.15 | 62.28 | 4.86 | 1.81 | 1.27 | 15.96 | 3.51 | 62.72 | 4.72 | 48.74 | 4.78 | 68.47 | 5.05 | 65.15 | 4.71 |
| 11 | 67.48 | 4.67 | 80.48 | 3.87 | 82.08 | 3.72 | 0.00 | 0.00 | 0.05 | 0.22 | 0.00 | 0.00 | 94.78 | 2.18 | 54.53 | 5.15 | 54.56 | 5.49 | 78.38 | 4.07 | 82.91 | 3.79 |
| 12 | 78.80 | 4.23 | 91.51 | 2.69 | 90.33 | 3.12 | 0.00 | 0.00 | 0.45 | 0.72 | 0.00 | 0.00 | 94.50 | 1.92 | 81.61 | 3.83 | 55.49 | 4.98 | 88.80 | 3.25 | 88.52 | 3.20 |
| 13 | 28.62 | 4.22 | 32.48 | 4.54 | 35.24 | 4.79 | 0.04 | 0.24 | 6.13 | 2.26 | 0.42 | 0.65 | 44.31 | 4.64 | 11.85 | 3.28 | 14.23 | 3.58 | 34.94 | 4.70 | 32.67 | 4.39 |
| 14 | 5.43 | 2.33 | 16.85 | 3.89 | 12.26 | 3.47 | 1.96 | 1.33 | 0.29 | 0.56 | 0.01 | 0.10 | 20.40 | 3.69 | 11.97 | 3.15 | 0.29 | 0.54 | 12.20 | 3.41 | 6.09 | 2.61 |
| 15 | 16.38 | 3.92 | 23.08 | 4.02 | 23.81 | 4.21 | 0.42 | 0.68 | 3.68 | 1.88 | 0.10 | 0.30 | 13.04 | 3.24 | 24.76 | 4.64 | 4.98 | 2.32 | 23.95 | 4.36 | 20.09 | 4.63 |
| 16 | 82.64 | 3.63 | 90.93 | 2.52 | 93.74 | 2.41 | 0.13 | 0.37 | 0.01 | 0.10 | 0.00 | 0.00 | 97.54 | 1.53 | 47.39 | 4.35 | 70.47 | 4.58 | 92.57 | 2.49 | 92.96 | 2.40 |
| 17 | 55.79 | 5.18 | 81.62 | 3.49 | 78.71 | 4.37 | 0.29 | 0.62 | 3.79 | 1.79 | 0.00 | 0.00 | 91.46 | 2.65 | 34.40 | 4.49 | 21.19 | 3.82 | 77.11 | 4.38 | 76.05 | 4.30 |
| 18 | 68.93 | 4.12 | 82.60 | 3.66 | 83.62 | 3.58 | 0.00 | 0.00 | 9.35 | 2.66 | 0.01 | 0.10 | 86.37 | 3.32 | 42.50 | 4.87 | 41.83 | 4.84 | 82.42 | 3.81 | 83.55 | 3.36 |
| 19 | 33.58 | 5.22 | 46.89 | 4.58 | 44.56 | 5.05 | 0.03 | 0.17 | 8.73 | 2.98 | 0.00 | 0.00 | 47.53 | 5.20 | 32.12 | 4.60 | 16.04 | 4.31 | 44.03 | 5.05 | 41.82 | 5.14 |
| 20 | 18.87 | 3.96 | 22.86 | 4.44 | 24.25 | 4.61 | 0.07 | 0.29 | 8.73 | 2.79 | 0.03 | 0.17 | 14.07 | 3.48 | 25.52 | 4.88 | 13.87 | 3.26 | 24.15 | 4.52 | 23.29 | 4.26 |
| 21 | 65.21 | 4.74 | 75.94 | 4.11 | 82.38 | 3.60 | 0.08 | 0.31 | 0.19 | 0.42 | 0.00 | 0.00 | 85.97 | 3.20 | 84.66 | 3.23 | 2.99 | 1.68 | 77.72 | 4.07 | 83.47 | 3.96 |
| 22 | 69.60 | 4.26 | 76.55 | 4.45 | 88.77 | 3.17 | 0.03 | 0.17 | 0.27 | 0.51 | 0.00 | 0.00 | 81.80 | 4.02 | 89.47 | 3.15 | 5.38 | 2.71 | 86.07 | 3.51 | 84.83 | 3.63 |
| 23 | 28.38 | 4.07 | 42.28 | 5.08 | 37.50 | 4.94 | 0.10 | 0.33 | 1.16 | 0.98 | 0.00 | 0.00 | 49.71 | 4.78 | 36.94 | 4.74 | 6.21 | 2.46 | 37.59 | 5.03 | 29.98 | 4.52 |
| 24 | 22.24 | 4.35 | 23.58 | 3.70 | 25.58 | 4.40 | 0.06 | 0.24 | 2.26 | 1.43 | 0.00 | 0.00 | 20.93 | 3.69 | 25.55 | 4.35 | 9.57 | 2.82 | 25.66 | 4.32 | 24.62 | 4.18 |
| 25 | 2.94 | 1.84 | 5.30 | 2.36 | 4.04 | 2.06 | 0.23 | 0.49 | 0.47 | 0.72 | 0.01 | 0.10 | 4.40 | 2.11 | 3.95 | 2.10 | 1.20 | 1.06 | 4.18 | 2.13 | 3.20 | 1.91 |
| 26 | 57.24 | 5.11 | 88.54 | 3.53 | 91.43 | 2.85 | 0.00 | 0.00 | 0.03 | 0.17 | 0.00 | 0.00 | 89.19 | 3.22 | 91.65 | 2.55 | 35.34 | 4.44 | 88.08 | 3.49 | 88.46 | 3.12 |
| 27 | 47.28 | 5.29 | 76.72 | 4.29 | 73.89 | 4.79 | 0.02 | 0.14 | 0.92 | 0.91 | 0.00 | 0.00 | 78.52 | 4.29 | 73.53 | 4.45 | 12.24 | 3.51 | 72.30 | 4.90 | 66.26 | 5.07 |
| 28 | 52.61 | 5.16 | 70.85 | 4.50 | 68.32 | 4.28 | 0.03 | 0.17 | 2.62 | 1.74 | 0.00 | 0.00 | 67.29 | 4.28 | 68.20 | 4.40 | 23.82 | 4.22 | 67.51 | 4.45 | 64.03 | 4.76 |
| 29 | 33.74 | 4.60 | 49.58 | 4.76 | 46.53 | 4.80 | 0.02 | 0.14 | 3.32 | 1.85 | 0.00 | 0.00 | 48.80 | 4.25 | 46.03 | 4.71 | 13.67 | 3.73 | 45.98 | 4.92 | 42.65 | 5.11 |
| 30 | 4.58 | 2.26 | 7.37 | 2.66 | 6.15 | 2.46 | 0.00 | 0.00 | 1.71 | 1.28 | 0.00 | 0.00 | 4.63 | 2.17 | 5.95 | 2.58 | 3.47 | 1.69 | 6.15 | 2.54 | 6.78 | 2.48 |
| 31 | 78.91 | 4.58 | 87.38 | 3.48 | 86.99 | 3.42 | 0.01 | 0.10 | 0.00 | 0.00 | 0.00 | 0.00 | 89.39 | 3.32 | 86.54 | 3.69 | 10.06 | 3.09 | 87.68 | 3.36 | 76.72 | 4.86 |
| 32 | 48.80 | 5.18 | 57.94 | 4.59 | 57.73 | 4.76 | 0.02 | 0.14 | 0.00 | 0.00 | 0.00 | 0.00 | 59.07 | 4.47 | 57.17 | 4.78 | 6.24 | 2.55 | 58.55 | 4.82 | 46.42 | 4.93 |
| 33 | 0.28 | 0.57 | 0.25 | 0.50 | 0.14 | 0.38 | 0.20 | 0.43 | 0.05 | 0.22 | 0.00 | 0.00 | 0.10 | 0.30 | 0.14 | 0.38 | 0.25 | 0.52 | 0.16 | 0.39 | 0.15 | 0.48 |
| 34 | 21.79 | 4.82 | 32.07 | 5.01 | 27.68 | 4.79 | 0.07 | 0.26 | 0.04 | 0.20 | 0.00 | 0.00 | 34.57 | 5.02 | 26.73 | 4.47 | 4.57 | 2.20 | 28.27 | 4.82 | 19.53 | 4.35 |
| 35 | 0.07 | 0.29 | 0.04 | 0.24 | 0.04 | 0.20 | 0.11 | 0.31 | 0.01 | 0.10 | 0.00 | 0.00 | 0.01 | 0.10 | 0.03 | 0.17 | 0.06 | 0.24 | 0.04 | 0.20 | 0.05 | 0.26 |
| 36 | 96.07 | 1.76 | 98.42 | 1.33 | 97.54 | 1.62 | 0.00 | 0.00 | 0.01 | 0.10 | 0.00 | 0.00 | 98.53 | 1.31 | 97.30 | 1.64 | 41.85 | 5.03 | 97.95 | 1.51 | 95.29 | 1.92 |
| 37 | 72.24 | 4.37 | 79.55 | 3.49 | 72.87 | 4.25 | 0.01 | 0.10 | 0.31 | 0.51 | 0.00 | 0.00 | 75.56 | 3.62 | 71.43 | 4.26 | 28.08 | 4.73 | 74.66 | 4.12 | 66.91 | 4.29 |
| 38 | 40.07 | 4.68 | 43.28 | 4.94 | 34.94 | 4.44 | 0.00 | 0.00 | 0.67 | 0.79 | 0.00 | 0.00 | 34.69 | 4.78 | 33.63 | 4.51 | 19.22 | 3.72 | 36.60 | 4.45 | 33.69 | 4.74 |
| 39 | 25.77 | 4.62 | 29.16 | 4.77 | 21.19 | 4.04 | 0.03 | 0.17 | 0.90 | 0.87 | 0.00 | 0.00 | 21.94 | 4.10 | 19.81 | 3.89 | 2.13 | 4.24 | 22.91 | 4.24 | 18.88 | 3.46 |
| 40 | 15.56 | 3.76 | 13.05 | 3.83 | 8.85 | 3.01 | 0.02 | 0.14 | 1.03 | 0.99 | 0.00 | 0.00 | 7.68 | 2.87 | 8.33 | 3.08 | 0.00 | 0.00 | 9.53 | 3.26 | 10.38 | 2.90 |

Supplemental Table 3. Power Results

| **Power** | | | | | | | | | | |
| --- | --- | --- | --- | --- | --- | --- | --- | --- | --- | --- |
|  | **CE** | | **LR** | | **NMI** | | **PSI** | | **YI** | |
| Model | Mean | Std Dev | Mean | Std Dev | Mean | Std Dev | Mean | Std Dev | Mean | Std Dev |
| 1 | 98.79 | 1.29 | 100.00 | 0.00 | 100.00 | 0.00 | 100.00 | 0.00 | 75.80 | 4.54 |
| 2 | 98.53 | 1.14 | 99.96 | 0.20 | 99.96 | 0.20 | 0.00 | 0.00 | 8.27 | 2.97 |
| 3 | 98.62 | 1.09 | 99.99 | 0.10 | 99.99 | 0.10 | 0.00 | 0.00 | 0.47 | 0.66 |
| 4 | 90.42 | 2.79 | 95.59 | 1.89 | 95.34 | 1.99 | 0.00 | 0.00 | 0.00 | 0.00 |
| 5 | 53.88 | 4.83 | 52.67 | 4.53 | 53.20 | 4.49 | 0.00 | 0.00 | 0.00 | 0.00 |
| 6 | 98.33 | 1.45 | 100.00 | 0.00 | 100.00 | 0.00 | 52.93 | 5.50 | 64.43 | 6.30 |
| 7 | 97.90 | 1.45 | 100.00 | 0.00 | 100.00 | 0.00 | 7.07 | 2.82 | 10.32 | 3.15 |
| 8 | 98.05 | 1.39 | 99.99 | 0.10 | 99.99 | 0.10 | 0.01 | 0.10 | 0.54 | 0.64 |
| 9 | 92.20 | 2.65 | 97.53 | 1.37 | 97.14 | 1.46 | 0.00 | 0.00 | 0.00 | 0.00 |
| 10 | 29.25 | 4.43 | 41.17 | 4.58 | 41.80 | 4.63 | 0.00 | 0.00 | 0.00 | 0.00 |
| 11 | 77.44 | 4.06 | 86.89 | 3.56 | 83.74 | 3.82 | 25.52 | 4.20 | 38.39 | 5.18 |
| 12 | 89.10 | 2.63 | 95.94 | 1.97 | 95.16 | 2.11 | 0.44 | 0.74 | 6.68 | 2.85 |
| 13 | 35.68 | 4.71 | 40.39 | 5.38 | 40.19 | 5.24 | 0.00 | 0.00 | 0.05 | 0.22 |
| 14 | 6.53 | 2.61 | 12.16 | 3.54 | 12.14 | 3.45 | 0.00 | 0.00 | 0.00 | 0.00 |
| 15 | 7.73 | 2.89 | 13.22 | 3.45 | 12.84 | 3.45 | 0.00 | 0.00 | 0.00 | 0.00 |
| 16 | 92.37 | 3.07 | 95.25 | 2.27 | 94.34 | 2.39 | 84.78 | 3.29 | 38.96 | 5.34 |
| 17 | 67.31 | 4.76 | 86.33 | 3.57 | 85.38 | 3.52 | 0.01 | 0.10 | 3.24 | 1.96 |
| 18 | 82.67 | 3.45 | 89.67 | 2.98 | 89.26 | 3.11 | 0.00 | 0.00 | 0.05 | 0.22 |
| 19 | 35.35 | 4.49 | 42.03 | 4.94 | 42.59 | 4.86 | 0.00 | 0.00 | 0.00 | 0.00 |
| 20 | 15.10 | 3.94 | 13.21 | 3.66 | 12.63 | 3.45 | 0.00 | 0.00 | 0.00 | 0.00 |
| 21 | 75.91 | 4.05 | 85.75 | 3.42 | 81.58 | 3.85 | 11.02 | 2.82 | 17.72 | 4.01 |
| 22 | 79.66 | 3.90 | 92.14 | 2.71 | 89.95 | 2.98 | 0.89 | 0.92 | 4.27 | 2.03 |
| 23 | 27.76 | 4.22 | 36.54 | 5.19 | 36.44 | 5.19 | 0.00 | 0.00 | 0.01 | 0.10 |
| 24 | 17.39 | 3.96 | 20.52 | 3.92 | 20.37 | 4.02 | 0.00 | 0.00 | 0.00 | 0.00 |
| 25 | 1.97 | 1.29 | 2.49 | 1.65 | 2.52 | 1.67 | 0.00 | 0.00 | 0.00 | 0.00 |
| 26 | 71.09 | 4.36 | 96.00 | 2.04 | 94.52 | 2.46 | 0.87 | 0.92 | 12.18 | 3.78 |
| 27 | 60.79 | 4.98 | 79.38 | 4.38 | 79.26 | 4.33 | 0.00 | 0.00 | 2.37 | 1.44 |
| 28 | 53.27 | 4.59 | 59.58 | 4.95 | 60.07 | 4.87 | 0.00 | 0.00 | 0.01 | 0.10 |
| 29 | 40.61 | 4.38 | 40.93 | 4.25 | 41.39 | 4.21 | 0.00 | 0.00 | 0.00 | 0.00 |
| 30 | 6.61 | 2.45 | 2.43 | 1.62 | 2.70 | 1.69 | 0.00 | 0.00 | 0.00 | 0.00 |
| 31 | 81.38 | 4.41 | 87.00 | 3.41 | 87.66 | 3.37 | 0.41 | 0.62 | 3.40 | 1.69 |
| 32 | 59.13 | 4.75 | 57.66 | 4.79 | 58.47 | 4.85 | 0.00 | 0.00 | 0.27 | 0.53 |
| 33 | 0.05 | 0.22 | 0.04 | 0.24 | 0.04 | 0.24 | 0.00 | 0.00 | 0.00 | 0.00 |
| 34 | 22.25 | 4.69 | 25.97 | 4.61 | 26.52 | 4.66 | 0.00 | 0.00 | 0.00 | 0.00 |
| 35 | 0.00 | 0.00 | 0.00 | 0.00 | 0.00 | 0.00 | 0.00 | 0.00 | 0.00 | 0.00 |
| 36 | 95.48 | 1.76 | 97.20 | 1.62 | 97.71 | 1.55 | 0.34 | 0.55 | 1.94 | 1.36 |
| 37 | 65.47 | 4.92 | 59.58 | 4.49 | 62.66 | 4.56 | 0.00 | 0.00 | 0.17 | 0.38 |
| 38 | 28.03 | 4.20 | 18.23 | 3.72 | 20.58 | 4.05 | 0.00 | 0.00 | 0.00 | 0.00 |
| 39 | 15.98 | 3.70 | 8.52 | 2.78 | 9.77 | 2.93 | 0.00 | 0.00 | 0.00 | 0.00 |
| 40 | 6.44 | 2.53 | 1.73 | 1.31 | 2.03 | 1.38 | 0.00 | 0.00 | 0.00 | 0.00 |

Supplemental Table 4. Specific Power Results

| **Specific Power** | | | | | | | | | | |
| --- | --- | --- | --- | --- | --- | --- | --- | --- | --- | --- |
|  | **CE** | | **LR** | | **NMI** | | **PSI** | | **YI** | |
| Model | Mean | Std Dev | Mean | Std Dev | Mean | Std Dev | Mean | Std Dev | Mean | Std Dev |
| 1 | 86.99 | 2.98 | 96.06 | 1.95 | 95.65 | 1.98 | 95.69 | 1.84 | 34.89 | 5.39 |
| 2 | 89.21 | 3.44 | 92.83 | 2.34 | 92.52 | 2.31 | 0.00 | 0.00 | 3.89 | 2.08 |
| 3 | 88.44 | 2.76 | 93.92 | 2.55 | 93.65 | 2.66 | 0.00 | 0.00 | 0.39 | 0.58 |
| 4 | 80.52 | 3.95 | 87.51 | 3.06 | 87.08 | 3.04 | 0.00 | 0.00 | 0.00 | 0.00 |
| 5 | 46.98 | 4.66 | 46.11 | 4.59 | 46.36 | 4.54 | 0.00 | 0.00 | 0.00 | 0.00 |
| 6 | 89.54 | 2.84 | 95.60 | 2.16 | 95.42 | 2.25 | 50.60 | 5.70 | 31.57 | 5.27 |
| 7 | 87.85 | 3.36 | 94.42 | 2.02 | 94.31 | 2.07 | 6.31 | 2.62 | 5.97 | 2.66 |
| 8 | 88.59 | 3.11 | 94.60 | 2.11 | 94.41 | 2.11 | 0.00 | 0.00 | 0.47 | 0.63 |
| 9 | 82.23 | 3.94 | 91.09 | 2.90 | 90.54 | 2.93 | 0.00 | 0.00 | 0.00 | 0.00 |
| 10 | 18.18 | 4.01 | 32.81 | 4.33 | 32.96 | 4.36 | 0.00 | 0.00 | 0.00 | 0.00 |
| 11 | 66.05 | 4.76 | 82.08 | 3.72 | 78.38 | 4.07 | 23.47 | 4.14 | 30.98 | 5.10 |
| 12 | 77.11 | 4.23 | 90.33 | 3.12 | 88.80 | 3.25 | 0.41 | 0.70 | 5.08 | 2.48 |
| 13 | 26.44 | 4.08 | 34.38 | 4.77 | 34.02 | 4.68 | 0.00 | 0.00 | 0.05 | 0.22 |
| 14 | 3.76 | 1.94 | 9.48 | 3.09 | 9.37 | 3.04 | 0.00 | 0.00 | 0.00 | 0.00 |
| 15 | 4.98 | 2.36 | 10.55 | 3.06 | 10.12 | 3.00 | 0.00 | 0.00 | 0.00 | 0.00 |
| 16 | 81.65 | 3.89 | 93.74 | 2.41 | 92.57 | 2.49 | 83.88 | 3.41 | 32.30 | 5.73 |
| 17 | 53.40 | 5.20 | 78.40 | 4.25 | 76.82 | 4.27 | 0.01 | 0.10 | 2.16 | 1.45 |
| 18 | 66.01 | 4.36 | 82.98 | 3.54 | 81.79 | 3.75 | 0.00 | 0.00 | 0.05 | 0.22 |
| 19 | 22.32 | 4.34 | 34.08 | 4.84 | 33.82 | 4.85 | 0.00 | 0.00 | 0.00 | 0.00 |
| 20 | 4.43 | 2.19 | 8.34 | 2.78 | 7.73 | 2.60 | 0.00 | 0.00 | 0.00 | 0.00 |
| 21 | 63.36 | 4.92 | 82.40 | 3.61 | 77.72 | 4.07 | 10.16 | 2.83 | 14.63 | 3.70 |
| 22 | 68.45 | 4.26 | 88.78 | 3.16 | 86.07 | 3.51 | 0.86 | 0.89 | 3.78 | 1.87 |
| 23 | 24.49 | 3.92 | 34.67 | 5.04 | 34.42 | 5.01 | 0.00 | 0.00 | 0.00 | 0.00 |
| 24 | 13.61 | 3.38 | 18.04 | 3.59 | 17.71 | 3.67 | 0.00 | 0.00 | 0.00 | 0.00 |
| 25 | 1.10 | 1.03 | 1.85 | 1.46 | 1.85 | 1.47 | 0.00 | 0.00 | 0.00 | 0.00 |
| 26 | 55.79 | 5.11 | 91.42 | 2.84 | 88.07 | 3.50 | 0.84 | 0.92 | 9.66 | 3.28 |
| 27 | 43.10 | 4.95 | 71.46 | 5.02 | 69.89 | 5.20 | 0.00 | 0.00 | 2.08 | 1.40 |
| 28 | 36.25 | 4.40 | 52.49 | 4.66 | 51.81 | 4.69 | 0.00 | 0.00 | 0.00 | 0.00 |
| 29 | 21.78 | 3.98 | 34.03 | 4.44 | 33.60 | 4.49 | 0.00 | 0.00 | 0.00 | 0.00 |
| 30 | 0.66 | 0.83 | 1.22 | 1.12 | 1.19 | 1.12 | 0.00 | 0.00 | 0.00 | 0.00 |
| 31 | 78.44 | 4.57 | 87.00 | 3.41 | 87.66 | 3.37 | 0.41 | 0.62 | 3.40 | 1.69 |
| 32 | 47.88 | 5.06 | 57.66 | 4.79 | 58.47 | 4.85 | 0.00 | 0.00 | 0.27 | 0.53 |
| 33 | 0.02 | 0.14 | 0.04 | 0.24 | 0.04 | 0.24 | 0.00 | 0.00 | 0.00 | 0.00 |
| 34 | 18.46 | 4.40 | 25.97 | 4.61 | 26.52 | 4.66 | 0.00 | 0.00 | 0.00 | 0.00 |
| 35 | 0.00 | 0.00 | 0.00 | 0.00 | 0.00 | 0.00 | 0.00 | 0.00 | 0.00 | 0.00 |
| 36 | 95.31 | 1.81 | 97.20 | 1.62 | 97.71 | 1.55 | 0.34 | 0.55 | 1.94 | 1.36 |
| 37 | 63.99 | 4.79 | 59.58 | 4.49 | 62.66 | 4.56 | 0.00 | 0.00 | 0.17 | 0.38 |
| 38 | 27.28 | 4.08 | 18.23 | 3.72 | 20.58 | 4.05 | 0.00 | 0.00 | 0.00 | 0.00 |
| 39 | 15.09 | 3.61 | 8.52 | 2.78 | 9.77 | 2.93 | 0.00 | 0.00 | 0.00 | 0.00 |
| 40 | 5.93 | 2.34 | 1.73 | 1.31 | 2.03 | 1.38 | 0.00 | 0.00 | 0.00 | 0.00 |

Supplemental Table 5. Two-Locus Penetrance Functions for Models 1-10

| **MAF** | **0.2** | | | | | **0.4** | | | | |
| --- | --- | --- | --- | --- | --- | --- | --- | --- | --- | --- |
| **Heritability** | **0.03** | **0.02** | **0.015** | **0.01** | **0.005** | **0.03** | **0.02** | **0.015** | **0.01** | **0.005** |
| **Model** | **1** | **2** | **3** | **4** | **5** | **6** | **7** | **8** | **9** | **10** |
| **aabb** | 0.0998 | 0.0786 | 0.0276 | 0.0884 | 0.0539 | 0.0848 | 0.0093 | 0.0381 | 0.0465 | 0.0161 |
| **Aabb** | 0.0984 | 0.0003 | 0.0942 | 0.0894 | 0.0732 | 0.0754 | 0.0281 | 0.0151 | 0.0368 | 0.0514 |
| **AAbb** | 0.0022 | 0.0967 | 0.0287 | 0.0307 | 0.0416 | 0.0053 | 0.0902 | 0.073 | 0.0706 | 0.0573 |
| **aaBb** | 0.0933 | 0.001 | 0.0941 | 0.071 | 0.007 | 0.0705 | 0.0491 | 0.0485 | 0.0666 | 0.0287 |
| **AaBb** | 0.0996 | 0.0013 | 0.0996 | 0.0036 | 0.0207 | 0.0135 | 0.0763 | 0.0618 | 0.0691 | 0.0442 |
| **AABb** | 0.0002 | 0.1001 | 0.0226 | 0.0737 | 0.0685 | 0.0967 | 0.0063 | 0.0067 | 0.02 | 0.0614 |
| **aaBB** | 0.0028 | 0.0948 | 0.0277 | 0.0368 | 0.0732 | 0.0118 | 0.0625 | 0.0288 | 0.0314 | 0.0867 |
| **AaBB** | 0 | 0.0998 | 0.0198 | 0.0711 | 0.066 | 0.0937 | 0.0161 | 0.0209 | 0.0329 | 0.0511 |
| **AABB** | 0.0574 | 0.0428 | 0.0657 | 0.0404 | 0.044 | 0.0131 | 0.0824 | 0.0693 | 0.0818 | 0.0253 |

Supplemental Table 6. Three-Locus Penetrance Functions for Models 11-20

| **MAF** | **0.2** | | | | | **0.4** | | | | |
| --- | --- | --- | --- | --- | --- | --- | --- | --- | --- | --- |
| **Heritability** | **0.03** | **0.02** | **0.015** | **0.01** | **0.005** | **0.03** | **0.02** | **0.015** | **0.01** | **0.005** |
| **Model** | **11** | **12** | **13** | **14** | **15** | **16** | **17** | **18** | **19** | **20** |
| **aabbcc** | 0.05 | 0 | 0.05 | 0.09 | 0.04 | 0.01 | 0.04 | 0.04 | 0.08 | 0.03 |
| **aabbCc** | 0.1 | 0.06 | 0.01 | 0.07 | 0.05 | 0.02 | 0.06 | 0.05 | 0.04 | 0.04 |
| **aabbCC** | 0.08 | 0.05 | 0.01 | 0.1 | 0.01 | 0.05 | 0.04 | 0.09 | 0.06 | 0.08 |
| **aaBbcc** | 0.02 | 0.05 | 0.08 | 0.01 | 0 | 0 | 0 | 0.06 | 0.01 | 0.04 |
| **aaBbCc** | 0.01 | 0.07 | 0.06 | 0.06 | 0.08 | 0.1 | 0.09 | 0.08 | 0.06 | 0.03 |
| **aaBbCC** | 0.01 | 0.04 | 0.03 | 0.01 | 0.04 | 0.09 | 0.07 | 0.03 | 0.05 | 0.05 |
| **aaBBcc** | 0.1 | 0.06 | 0.06 | 0.07 | 0.07 | 0.02 | 0.01 | 0.05 | 0.08 | 0.09 |
| **aaBBCc** | 0.06 | 0 | 0.05 | 0.07 | 0.05 | 0 | 0.07 | 0.02 | 0.08 | 0.06 |
| **aaBBCC** | 0.05 | 0.06 | 0.06 | 0.09 | 0.04 | 0.08 | 0.02 | 0.03 | 0.1 | 0.05 |
| **aAbbcc** | 0.01 | 0.01 | 0.05 | 0.01 | 0 | 0.09 | 0.04 | 0.03 | 0.02 | 0.07 |
| **aAbbCc** | 0.08 | 0.09 | 0.02 | 0 | 0.08 | 0.1 | 0.1 | 0.02 | 0.05 | 0.03 |
| **aAbbCC** | 0.06 | 0.06 | 0.09 | 0.06 | 0.05 | 0.01 | 0.04 | 0.09 | 0.08 | 0.03 |
| **aABbcc** | 0.07 | 0.01 | 0.09 | 0.03 | 0.09 | 0 | 0.05 | 0.05 | 0.09 | 0.04 |
| **aABbCc** | 0.03 | 0.06 | 0.07 | 0.06 | 0 | 0.1 | 0 | 0.02 | 0.05 | 0.05 |
| **aABbCC** | 0.07 | 0 | 0.1 | 0.09 | 0.08 | 0.01 | 0.08 | 0.09 | 0.04 | 0.06 |
| **aABBcc** | 0.06 | 0.04 | 0.03 | 0.09 | 0.02 | 0.09 | 0.1 | 0.07 | 0.03 | 0.06 |
| **aABBCc** | 0.09 | 0.03 | 0.02 | 0 | 0.04 | 0 | 0.09 | 0.07 | 0.08 | 0.05 |
| **aABBCC** | 0 | 0.07 | 0.03 | 0.09 | 0.04 | 0.1 | 0.05 | 0.01 | 0.1 | 0.05 |
| **AAbbcc** | 0 | 0.01 | 0.09 | 0.03 | 0.01 | 0.05 | 0.05 | 0.08 | 0.08 | 0.03 |
| **AAbbCc** | 0.07 | 0.08 | 0.03 | 0.1 | 0.07 | 0.08 | 0.04 | 0.02 | 0.06 | 0.08 |
| **AAbbCC** | 0.01 | 0.01 | 0.05 | 0.07 | 0.03 | 0 | 0.05 | 0.06 | 0.1 | 0.06 |
| **AABbcc** | 0.02 | 0 | 0.1 | 0.08 | 0.07 | 0.08 | 0.05 | 0.04 | 0.08 | 0.08 |
| **AABbCc** | 0.08 | 0 | 0 | 0.1 | 0.06 | 0 | 0.1 | 0.05 | 0.09 | 0.04 |
| **AABbCC** | 0.02 | 0.09 | 0.04 | 0.04 | 0.03 | 0.1 | 0.06 | 0.03 | 0.08 | 0.05 |
| **AABBcc** | 0.04 | 0.08 | 0.02 | 0.06 | 0.04 | 0.1 | 0.09 | 0.02 | 0.08 | 0 |
| **AABBCc** | 0 | 0.07 | 0.09 | 0.08 | 0.05 | 0.09 | 0.01 | 0.09 | 0.04 | 0.07 |
| **AABBCC** | 0.07 | 0.02 | 0.05 | 0.06 | 0.05 | 0.02 | 0.04 | 0.03 | 0 | 0.03 |

Supplemental Table 7. Four-Locus Penetrance Functions for Models 21-30

| **MAF** | **0.2** | | | | | **0.4** | | | | |
| --- | --- | --- | --- | --- | --- | --- | --- | --- | --- | --- |
| **Heritability** | **0.03** | **0.02** | **0.015** | **0.01** | **0.005** | **0.03** | **0.02** | **0.015** | **0.01** | **0.005** |
| **Model** | 21 | 22 | 23 | 24 | 25 | 26 | 27 | 28 | 29 | 30 |
| **aabbccdd** | 0.07 | 0.02 | 0.07 | 0.05 | 0.05 | 0.04 | 0.03 | 0 | 0.02 | 0.02 |
| **aabbCcdd** | 0.09 | 0.02 | 0.01 | 0.06 | 0.03 | 0.04 | 0.08 | 0.07 | 0.03 | 0.03 |
| **aabbCCdd** | 0.02 | 0 | 0 | 0.1 | 0.02 | 0.03 | 0.1 | 0.02 | 0.02 | 0.01 |
| **aaBbccdd** | 0.04 | 0.09 | 0.1 | 0.09 | 0.03 | 0.02 | 0.02 | 0.04 | 0.08 | 0.1 |
| **aaBbCcdd** | 0.02 | 0.09 | 0.01 | 0.06 | 0.03 | 0 | 0.06 | 0.01 | 0.02 | 0.05 |
| **aaBbCCdd** | 0 | 0.1 | 0.01 | 0.1 | 0.04 | 0.06 | 0.07 | 0.08 | 0.02 | 0.02 |
| **aaBBccdd** | 0.05 | 0.01 | 0.04 | 0.07 | 0.04 | 0.01 | 0 | 0.06 | 0.01 | 0.03 |
| **aaBBCcdd** | 0.07 | 0.03 | 0.07 | 0.07 | 0.02 | 0.04 | 0.09 | 0.06 | 0.02 | 0.1 |
| **aaBBCCdd** | 0.1 | 0.08 | 0.04 | 0.07 | 0.03 | 0.05 | 0.09 | 0 | 0.04 | 0.06 |
| **aAbbccdd** | 0 | 0.04 | 0.09 | 0.07 | 0.04 | 0 | 0.09 | 0.08 | 0.03 | 0.08 |
| **aAbbCcdd** | 0.04 | 0.07 | 0.04 | 0.07 | 0.06 | 0.02 | 0.05 | 0.1 | 0.1 | 0.08 |
| **aAbbCCdd** | 0.01 | 0.05 | 0.1 | 0.03 | 0.07 | 0.04 | 0.03 | 0 | 0.06 | 0.09 |
| **aABbccdd** | 0 | 0 | 0.07 | 0.07 | 0.06 | 0.01 | 0.03 | 0.1 | 0.04 | 0.07 |
| **aABbCcdd** | 0.1 | 0.1 | 0.01 | 0.09 | 0.03 | 0.1 | 0.07 | 0.08 | 0.06 | 0.05 |
| **aABbCCdd** | 0.01 | 0 | 0.06 | 0.03 | 0.06 | 0.04 | 0.09 | 0 | 0.02 | 0.02 |
| **aABBccdd** | 0.02 | 0 | 0.1 | 0 | 0.03 | 0 | 0.01 | 0.1 | 0.04 | 0.07 |
| **aABBCcdd** | 0.01 | 0.06 | 0.1 | 0.04 | 0.06 | 0.07 | 0.06 | 0.02 | 0.05 | 0.05 |
| **aABBCCdd** | 0.01 | 0.05 | 0.03 | 0.09 | 0.06 | 0 | 0.01 | 0.1 | 0.07 | 0.06 |
| **AAbbccdd** | 0 | 0 | 0.1 | 0.06 | 0.04 | 0.03 | 0.06 | 0.09 | 0.06 | 0.05 |
| **AAbbCcdd** | 0.09 | 0.04 | 0.08 | 0.09 | 0.03 | 0.08 | 0 | 0.07 | 0.1 | 0.04 |
| **AAbbCCdd** | 0 | 0.08 | 0.03 | 0.07 | 0.05 | 0.04 | 0 | 0.02 | 0.08 | 0.02 |
| **AABbccdd** | 0.02 | 0.1 | 0.03 | 0.06 | 0.06 | 0.07 | 0.05 | 0.06 | 0.04 | 0.05 |
| **AABbCcdd** | 0.04 | 0.05 | 0.09 | 0.05 | 0.04 | 0.07 | 0.03 | 0.07 | 0.02 | 0.06 |
| **AABbCCdd** | 0.04 | 0.07 | 0.03 | 0.05 | 0.03 | 0 | 0.05 | 0.01 | 0 | 0.06 |
| **AABBccdd** | 0.09 | 0.1 | 0.1 | 0.06 | 0.04 | 0.02 | 0.05 | 0.03 | 0.1 | 0.04 |
| **AABBCcdd** | 0.06 | 0.03 | 0.02 | 0.07 | 0 | 0.01 | 0.05 | 0.08 | 0.06 | 0.04 |
| **AABBCCdd** | 0.07 | 0.01 | 0.07 | 0.02 | 0.06 | 0.09 | 0.08 | 0.05 | 0.02 | 0.05 |
| **aabbccDd** | 0.07 | 0 | 0.06 | 0.08 | 0.03 | 0.1 | 0.1 | 0.09 | 0.06 | 0.04 |
| **aabbCcDd** | 0.08 | 0.1 | 0.06 | 0.09 | 0.02 | 0.07 | 0.04 | 0.08 | 0.04 | 0.07 |
| **aabbCCDd** | 0.03 | 0.1 | 0.1 | 0.09 | 0.03 | 0.09 | 0.1 | 0.1 | 0.01 | 0.03 |
| **aaBbccDd** | 0 | 0 | 0.07 | 0.08 | 0.02 | 0.1 | 0.07 | 0.02 | 0.01 | 0.05 |
| **aaBbCcDd** | 0 | 0.09 | 0 | 0 | 0.01 | 0.03 | 0 | 0.07 | 0.07 | 0.04 |
| **aaBbCCDd** | 0.01 | 0.01 | 0.08 | 0.08 | 0.02 | 0.03 | 0.09 | 0.05 | 0.04 | 0.1 |
| **aaBBccDd** | 0.03 | 0.1 | 0.06 | 0.01 | 0.03 | 0.07 | 0.01 | 0.09 | 0.09 | 0.06 |
| **aaBBCcDd** | 0.06 | 0.07 | 0.04 | 0.02 | 0.01 | 0.03 | 0.08 | 0.08 | 0.07 | 0.06 |
| **aaBBCCDd** | 0.06 | 0.08 | 0.01 | 0.06 | 0.02 | 0.03 | 0.08 | 0.09 | 0.04 | 0.07 |
| **aAbbccDd** | 0 | 0.07 | 0.09 | 0.06 | 0.04 | 0.1 | 0 | 0.01 | 0 | 0.08 |
| **aAbbCcDd** | 0.03 | 0.06 | 0.09 | 0.08 | 0.04 | 0.1 | 0 | 0.02 | 0 | 0.03 |
| **aAbbCCDd** | 0.05 | 0.01 | 0.09 | 0.06 | 0.05 | 0.02 | 0.08 | 0.09 | 0 | 0.06 |
| **aABbccDd** | 0.01 | 0.03 | 0.01 | 0.06 | 0.04 | 0.02 | 0.05 | 0.03 | 0.06 | 0.05 |
| **aABbCcDd** | 0.02 | 0.05 | 0.1 | 0.07 | 0.01 | 0 | 0.08 | 0.07 | 0.03 | 0.04 |
| **aABbCCDd** | 0 | 0.08 | 0.1 | 0.01 | 0.05 | 0.03 | 0.04 | 0.05 | 0.06 | 0.04 |
| **aABBccDd** | 0.09 | 0.04 | 0.02 | 0.06 | 0.03 | 0 | 0.05 | 0.05 | 0.01 | 0.05 |
| **aABBCcDd** | 0.04 | 0.08 | 0.02 | 0.06 | 0.03 | 0.1 | 0.05 | 0.04 | 0.05 | 0.05 |
| **aABBCCDd** | 0 | 0.06 | 0.08 | 0.01 | 0.03 | 0.01 | 0.06 | 0.03 | 0.03 | 0.06 |
| **AAbbccDd** | 0.07 | 0 | 0.1 | 0.05 | 0.03 | 0.01 | 0.09 | 0.05 | 0.01 | 0.03 |
| **AAbbCcDd** | 0.05 | 0.08 | 0.01 | 0.1 | 0.08 | 0 | 0.08 | 0.1 | 0.03 | 0.06 |
| **AAbbCCDd** | 0.04 | 0.03 | 0.03 | 0.05 | 0.03 | 0.01 | 0.02 | 0.02 | 0.07 | 0.03 |
| **AABbccDd** | 0 | 0.04 | 0 | 0.1 | 0.03 | 0.02 | 0.04 | 0.07 | 0.02 | 0.07 |
| **AABbCcDd** | 0.02 | 0 | 0.08 | 0.03 | 0.07 | 0.1 | 0.03 | 0.02 | 0.06 | 0.06 |
| **AABbCCDd** | 0.04 | 0 | 0.03 | 0.05 | 0.05 | 0.06 | 0.04 | 0.05 | 0.08 | 0.06 |
| **AABBccDd** | 0.07 | 0.08 | 0.1 | 0.05 | 0.04 | 0.09 | 0.09 | 0.04 | 0.09 | 0.05 |
| **AABBCcDd** | 0.06 | 0.01 | 0.02 | 0.05 | 0.03 | 0 | 0.03 | 0.07 | 0.05 | 0.05 |
| **AABBCCDd** | 0.08 | 0.05 | 0.05 | 0.07 | 0.06 | 0.08 | 0.09 | 0.07 | 0.02 | 0.05 |
| **aabbccDD** | 0.07 | 0.04 | 0.03 | 0.01 | 0.04 | 0.05 | 0.03 | 0.03 | 0.07 | 0.04 |
| **aabbCcDD** | 0.04 | 0 | 0 | 0.05 | 0.03 | 0.04 | 0.07 | 0 | 0.05 | 0.06 |
| **aabbCCDD** | 0.08 | 0.06 | 0.1 | 0.06 | 0.04 | 0.02 | 0.1 | 0 | 0.1 | 0.02 |
| **aaBbccDD** | 0.03 | 0.1 | 0.02 | 0 | 0.02 | 0.09 | 0.01 | 0.04 | 0.05 | 0 |
| **aaBbCcDD** | 0.08 | 0.03 | 0 | 0.05 | 0.01 | 0.04 | 0 | 0.05 | 0.03 | 0.06 |
| **aaBbCCDD** | 0.03 | 0.04 | 0.02 | 0.08 | 0.07 | 0.04 | 0.07 | 0.07 | 0.04 | 0.04 |
| **aaBBccDD** | 0.06 | 0.08 | 0 | 0.06 | 0 | 0.1 | 0.03 | 0.1 | 0.04 | 0.06 |
| **aaBBCcDD** | 0.09 | 0.01 | 0.07 | 0.02 | 0.08 | 0.01 | 0.06 | 0 | 0.04 | 0.03 |
| **aaBBCCDD** | 0.02 | 0.02 | 0.1 | 0.05 | 0.06 | 0.07 | 0.01 | 0.02 | 0.02 | 0.03 |
| **aAbbccDD** | 0.06 | 0 | 0.07 | 0.04 | 0.04 | 0.01 | 0 | 0.05 | 0.1 | 0.07 |
| **aAbbCcDD** | 0.01 | 0.07 | 0.07 | 0.07 | 0.06 | 0.06 | 0.1 | 0.02 | 0.07 | 0.07 |
| **aAbbCCDD** | 0.09 | 0 | 0.08 | 0.05 | 0.03 | 0.01 | 0.04 | 0.07 | 0.03 | 0.04 |
| **aABbccDD** | 0.08 | 0 | 0 | 0.01 | 0.07 | 0.06 | 0.1 | 0.09 | 0.05 | 0.07 |
| **aABbCcDD** | 0 | 0.06 | 0.09 | 0.06 | 0.07 | 0.02 | 0.1 | 0.07 | 0.05 | 0.06 |
| **aABbCCDD** | 0.1 | 0.07 | 0.08 | 0.1 | 0.05 | 0.1 | 0 | 0.03 | 0.05 | 0.06 |
| **aABBccDD** | 0.02 | 0.09 | 0.09 | 0.02 | 0.07 | 0.05 | 0.03 | 0.05 | 0.06 | 0.02 |
| **aABBCcDD** | 0.04 | 0 | 0.02 | 0.03 | 0.03 | 0.08 | 0 | 0.06 | 0.04 | 0.05 |
| **aABBCCDD** | 0.08 | 0.01 | 0.02 | 0.06 | 0.05 | 0.04 | 0.06 | 0.08 | 0.06 | 0.06 |
| **AAbbccDD** | 0 | 0.05 | 0 | 0.04 | 0.05 | 0.07 | 0.07 | 0.07 | 0.04 | 0.08 |
| **AAbbCcDD** | 0.03 | 0 | 0.01 | 0 | 0.03 | 0.01 | 0.07 | 0.1 | 0.07 | 0.05 |
| **AAbbCCDD** | 0.04 | 0.07 | 0.06 | 0.05 | 0.05 | 0.09 | 0.05 | 0.03 | 0.06 | 0.06 |
| **AABbccDD** | 0.09 | 0.04 | 0.07 | 0.04 | 0.04 | 0.04 | 0.06 | 0.02 | 0.03 | 0.02 |
| **AABbCcDD** | 0 | 0.1 | 0.02 | 0.02 | 0.06 | 0.02 | 0.08 | 0.05 | 0.01 | 0.06 |
| **AABbCCDD** | 0.08 | 0.01 | 0.04 | 0.05 | 0.02 | 0.05 | 0.01 | 0.1 | 0.05 | 0.04 |
| **AABBccDD** | 0.02 | 0 | 0.04 | 0.07 | 0.04 | 0.05 | 0.1 | 0.04 | 0.03 | 0.05 |
| **AABBCcDD** | 0.07 | 0.04 | 0.09 | 0.08 | 0.05 | 0.01 | 0.04 | 0.01 | 0.03 | 0.05 |
| **AABBCCDD** | 0 | 0.06 | 0.06 | 0.04 | 0.04 | 0.01 | 0.1 | 0.07 | 0.04 | 0.06 |

Supplemental Table 8. Five-Locus Penetrance Tables for Models 31-40

| **MAF** | **0.2** | | | | | **0.4** | | | | |
| --- | --- | --- | --- | --- | --- | --- | --- | --- | --- | --- |
| **Heritability** | **0.03** | **0.02** | **0.015** | **0.01** | **0.005** | **0.03** | **0.02** | **0.015** | **0.01** | **0.005** |
| **Model** | 31 | 32 | 33 | 34 | 35 | 36 | 37 | 38 | 39 | 40 |
| **aabbccddee** | 0.05 | 0.02 | 0.03 | 0.09 | 0.08 | 0.07 | 0.1 | 0.05 | 0.09 | 0.04 |
| **aabbCcddee** | 0.09 | 0.06 | 0.03 | 0.03 | 0.09 | 0.09 | 0.03 | 0 | 0.08 | 0.09 |
| **aabbCCddee** | 0.09 | 0 | 0.01 | 0.01 | 0.05 | 0.03 | 0 | 0.09 | 0.06 | 0.05 |
| **aaBbccddee** | 0 | 0.1 | 0.02 | 0.08 | 0.01 | 0.05 | 0.01 | 0.08 | 0.03 | 0.06 |
| **aaBbCcddee** | 0.05 | 0.01 | 0.06 | 0 | 0.09 | 0.05 | 0.05 | 0.07 | 0.01 | 0.03 |
| **aaBbCCddee** | 0.1 | 0.05 | 0.09 | 0.09 | 0.06 | 0.01 | 0.05 | 0 | 0 | 0 |
| **aaBBccddee** | 0.06 | 0.09 | 0.1 | 0.02 | 0.01 | 0.1 | 0.08 | 0.05 | 0.04 | 0.09 |
| **aaBBCcddee** | 0 | 0.05 | 0 | 0.06 | 0.07 | 0 | 0.1 | 0.1 | 0.06 | 0.06 |
| **aaBBCCddee** | 0.02 | 0.06 | 0.1 | 0.02 | 0.03 | 0.01 | 0.1 | 0.07 | 0.06 | 0.07 |
| **aAbbccddee** | 0 | 0.03 | 0.07 | 0.03 | 0.09 | 0.05 | 0.05 | 0.09 | 0.01 | 0.03 |
| **aAbbCcddee** | 0.05 | 0.03 | 0.05 | 0.05 | 0.05 | 0.08 | 0 | 0 | 0.07 | 0.07 |
| **aAbbCCddee** | 0.01 | 0.09 | 0.09 | 0.01 | 0.02 | 0.1 | 0.09 | 0.09 | 0.1 | 0.03 |
| **aABbccddee** | 0.02 | 0.05 | 0.1 | 0.02 | 0.09 | 0.02 | 0.1 | 0.02 | 0.04 | 0.02 |
| **aABbCcddee** | 0.01 | 0.04 | 0.09 | 0.04 | 0.08 | 0.02 | 0.03 | 0.02 | 0.05 | 0.04 |
| **aABbCCddee** | 0.04 | 0.05 | 0.09 | 0.06 | 0.04 | 0.1 | 0.09 | 0.02 | 0.01 | 0.02 |
| **aABBccddee** | 0.04 | 0.08 | 0.03 | 0.09 | 0.05 | 0.09 | 0.08 | 0.05 | 0.06 | 0.07 |
| **aABBCcddee** | 0.06 | 0.09 | 0.09 | 0.07 | 0.05 | 0.08 | 0.06 | 0.03 | 0.04 | 0.07 |
| **aABBCCddee** | 0.1 | 0.06 | 0.04 | 0.06 | 0.05 | 0.02 | 0.09 | 0 | 0 | 0.05 |
| **AAbbccddee** | 0.09 | 0.02 | 0.07 | 0.08 | 0.05 | 0.1 | 0.01 | 0.04 | 0.02 | 0.02 |
| **AAbbCcddee** | 0.03 | 0.09 | 0.1 | 0.03 | 0.03 | 0.05 | 0.04 | 0.01 | 0.02 | 0.06 |
| **AAbbCCddee** | 0.1 | 0.07 | 0.05 | 0.05 | 0.1 | 0.08 | 0.08 | 0.02 | 0.07 | 0.03 |
| **AABbccddee** | 0.09 | 0.03 | 0.06 | 0.02 | 0.03 | 0.05 | 0.1 | 0.03 | 0 | 0.07 |
| **AABbCcddee** | 0.09 | 0.01 | 0.09 | 0.04 | 0.03 | 0.01 | 0.09 | 0.03 | 0.03 | 0 |
| **AABbCCddee** | 0.06 | 0.04 | 0.08 | 0.08 | 0.02 | 0 | 0.03 | 0.06 | 0.08 | 0.03 |
| **AABBccddee** | 0.01 | 0.02 | 0.05 | 0 | 0.05 | 0.06 | 0 | 0.06 | 0.08 | 0.06 |
| **AABBCcddee** | 0.02 | 0 | 0.03 | 0.07 | 0.05 | 0.03 | 0.01 | 0.03 | 0.01 | 0.08 |
| **AABBCCddee** | 0.08 | 0.06 | 0.04 | 0.06 | 0.04 | 0.05 | 0.03 | 0.01 | 0.03 | 0.04 |
| **aabbccDdee** | 0.02 | 0 | 0.07 | 0.05 | 0.02 | 0.09 | 0.02 | 0 | 0.08 | 0.07 |
| **aabbCcDdee** | 0 | 0.07 | 0.03 | 0.05 | 0.08 | 0.04 | 0.07 | 0.03 | 0.05 | 0.06 |
| **aabbCCDdee** | 0.1 | 0.05 | 0.04 | 0.06 | 0.06 | 0.05 | 0.08 | 0.05 | 0.08 | 0.09 |
| **aaBbccDdee** | 0.01 | 0 | 0.09 | 0.03 | 0.05 | 0.09 | 0.05 | 0.03 | 0.03 | 0.02 |
| **aaBbCcDdee** | 0.05 | 0.03 | 0.09 | 0.08 | 0.04 | 0.09 | 0.04 | 0.09 | 0.02 | 0.04 |
| **aaBbCCDdee** | 0 | 0.05 | 0.03 | 0.1 | 0.03 | 0.1 | 0.09 | 0.03 | 0.08 | 0.04 |
| **aaBBccDdee** | 0.02 | 0.05 | 0.03 | 0 | 0.08 | 0.08 | 0.1 | 0.02 | 0.1 | 0.05 |
| **aaBBCcDdee** | 0.05 | 0.08 | 0.09 | 0.07 | 0.05 | 0.05 | 0.01 | 0.07 | 0.01 | 0.01 |
| **aaBBCCDdee** | 0.01 | 0.05 | 0.04 | 0.03 | 0.03 | 0.06 | 0.03 | 0.1 | 0.05 | 0.08 |
| **aAbbccDdee** | 0.07 | 0.03 | 0.09 | 0.07 | 0.01 | 0.05 | 0.07 | 0.01 | 0.02 | 0.04 |
| **aAbbCcDdee** | 0.08 | 0.06 | 0.02 | 0.05 | 0.05 | 0.08 | 0.06 | 0.02 | 0.07 | 0.06 |
| **aAbbCCDdee** | 0 | 0.07 | 0.02 | 0.06 | 0.04 | 0 | 0.1 | 0.02 | 0.03 | 0.09 |
| **aABbccDdee** | 0.04 | 0 | 0.04 | 0.1 | 0.05 | 0.02 | 0.03 | 0.03 | 0.07 | 0.04 |
| **aABbCcDdee** | 0.04 | 0 | 0.07 | 0.06 | 0.03 | 0.07 | 0.04 | 0.05 | 0.04 | 0.05 |
| **aABbCCDdee** | 0.05 | 0.01 | 0.06 | 0.03 | 0.05 | 0 | 0.02 | 0.02 | 0.08 | 0.05 |
| **aABBccDdee** | 0.08 | 0.01 | 0.03 | 0.01 | 0.05 | 0.05 | 0.09 | 0.09 | 0.05 | 0.07 |
| **aABBCcDdee** | 0.07 | 0.06 | 0.05 | 0.04 | 0.05 | 0.1 | 0.06 | 0.02 | 0.05 | 0.08 |
| **aABBCCDdee** | 0.04 | 0.01 | 0.02 | 0.08 | 0.04 | 0.1 | 0.06 | 0.04 | 0.06 | 0.04 |
| **AAbbccDdee** | 0.07 | 0.09 | 0.03 | 0.02 | 0.04 | 0.07 | 0 | 0.07 | 0.03 | 0.06 |
| **AAbbCcDdee** | 0.02 | 0 | 0.1 | 0.06 | 0.03 | 0.06 | 0.04 | 0.01 | 0.04 | 0.07 |
| **AAbbCCDdee** | 0.02 | 0.08 | 0.03 | 0.08 | 0.04 | 0.1 | 0 | 0.05 | 0.04 | 0.03 |
| **AABbccDdee** | 0.07 | 0.1 | 0.1 | 0.08 | 0.05 | 0 | 0.04 | 0 | 0.03 | 0.07 |
| **AABbCcDdee** | 0.06 | 0.09 | 0.05 | 0.02 | 0.05 | 0.1 | 0.06 | 0.07 | 0.04 | 0.06 |
| **AABbCCDdee** | 0.06 | 0.1 | 0.05 | 0.01 | 0.06 | 0 | 0.1 | 0.07 | 0.1 | 0.03 |
| **AABBccDdee** | 0 | 0.02 | 0.09 | 0.06 | 0.04 | 0.03 | 0.07 | 0.03 | 0.06 | 0.05 |
| **AABBCcDdee** | 0 | 0.09 | 0.01 | 0.04 | 0.05 | 0.1 | 0.05 | 0.04 | 0.06 | 0.06 |
| **AABBCCDdee** | 0.09 | 0.1 | 0.06 | 0.09 | 0.07 | 0.05 | 0.01 | 0.05 | 0.02 | 0.07 |
| **aabbccDDee** | 0 | 0.05 | 0.08 | 0.02 | 0.07 | 0.09 | 0.09 | 0.05 | 0.09 | 0.06 |
| **aabbCcDDee** | 0.07 | 0.1 | 0.04 | 0.06 | 0.08 | 0.03 | 0.04 | 0 | 0.03 | 0.04 |
| **aabbCCDDee** | 0.04 | 0.08 | 0.02 | 0.07 | 0.08 | 0.06 | 0 | 0.07 | 0.08 | 0.06 |
| **aaBbccDDee** | 0.1 | 0 | 0.04 | 0.03 | 0.08 | 0.06 | 0.09 | 0.07 | 0.04 | 0.05 |
| **aaBbCcDDee** | 0.09 | 0.04 | 0.1 | 0.03 | 0.05 | 0.03 | 0.06 | 0.03 | 0.02 | 0.07 |
| **aaBbCCDDee** | 0.01 | 0.01 | 0.06 | 0.04 | 0.06 | 0.03 | 0.02 | 0.09 | 0 | 0.06 |
| **aaBBccDDee** | 0.1 | 0.09 | 0.03 | 0.03 | 0.06 | 0.01 | 0.01 | 0 | 0.05 | 0.1 |
| **aaBBCcDDee** | 0.05 | 0.08 | 0.07 | 0.05 | 0.07 | 0.1 | 0.08 | 0.09 | 0.02 | 0.05 |
| **aaBBCCDDee** | 0.04 | 0.08 | 0.07 | 0.07 | 0.05 | 0.04 | 0.1 | 0.07 | 0.06 | 0.05 |
| **aAbbccDDee** | 0.01 | 0.07 | 0.05 | 0.07 | 0.06 | 0.09 | 0.05 | 0.08 | 0.04 | 0.04 |
| **aAbbCcDDee** | 0.05 | 0.08 | 0.1 | 0.02 | 0.02 | 0.01 | 0.09 | 0.02 | 0.1 | 0.05 |
| **aAbbCCDDee** | 0.03 | 0 | 0.1 | 0.1 | 0.04 | 0.01 | 0.03 | 0.02 | 0.04 | 0.08 |
| **aABbccDDee** | 0 | 0.01 | 0.06 | 0.03 | 0.06 | 0.01 | 0.08 | 0.1 | 0.05 | 0.06 |
| **aABbCcDDee** | 0 | 0.09 | 0.03 | 0.07 | 0.05 | 0 | 0.09 | 0.07 | 0.05 | 0.07 |
| **aABbCCDDee** | 0.02 | 0.01 | 0.07 | 0.02 | 0.04 | 0.1 | 0.04 | 0.08 | 0.07 | 0.06 |
| **aABBccDDee** | 0.09 | 0.03 | 0.1 | 0.02 | 0.05 | 0.05 | 0.09 | 0 | 0.07 | 0.03 |
| **aABBCcDDee** | 0 | 0.03 | 0.05 | 0.02 | 0.05 | 0.01 | 0.01 | 0.07 | 0.02 | 0.03 |
| **aABBCCDDee** | 0.09 | 0.03 | 0.07 | 0.04 | 0.06 | 0.07 | 0.08 | 0.09 | 0.02 | 0.05 |
| **AAbbccDDee** | 0.08 | 0.05 | 0.02 | 0 | 0.05 | 0.04 | 0 | 0.02 | 0.02 | 0 |
| **AAbbCcDDee** | 0.06 | 0.03 | 0 | 0.04 | 0.05 | 0.07 | 0.09 | 0.02 | 0.02 | 0.06 |
| **AAbbCCDDee** | 0.09 | 0.08 | 0.06 | 0.08 | 0.04 | 0.01 | 0.04 | 0.08 | 0.08 | 0.08 |
| **AABbccDDee** | 0.1 | 0.03 | 0.08 | 0.05 | 0.04 | 0.08 | 0.1 | 0 | 0.05 | 0.09 |
| **AABbCcDDee** | 0.1 | 0.01 | 0.07 | 0.06 | 0.04 | 0.06 | 0 | 0.04 | 0.07 | 0.06 |
| **AABbCCDDee** | 0.07 | 0.03 | 0.07 | 0.03 | 0.03 | 0.1 | 0.04 | 0 | 0.05 | 0.04 |
| **AABBccDDee** | 0.07 | 0.09 | 0.02 | 0.03 | 0.05 | 0 | 0.03 | 0.05 | 0.03 | 0.08 |
| **AABBCcDDee** | 0.09 | 0.05 | 0.09 | 0.1 | 0.05 | 0 | 0.02 | 0.08 | 0.07 | 0.06 |
| **AABBCCDDee** | 0.01 | 0.03 | 0 | 0.03 | 0.03 | 0 | 0.03 | 0.06 | 0.07 | 0.04 |
| **aabbccddEe** | 0.01 | 0.06 | 0.07 | 0.03 | 0.03 | 0.1 | 0.04 | 0.02 | 0.03 | 0.09 |
| **aabbCcddEe** | 0.1 | 0.01 | 0.06 | 0.01 | 0.01 | 0.09 | 0.09 | 0.04 | 0.02 | 0.04 |
| **aabbCCddEe** | 0.1 | 0.05 | 0.08 | 0.09 | 0.07 | 0.06 | 0 | 0.06 | 0.06 | 0.02 |
| **aaBbccddEe** | 0 | 0.07 | 0.09 | 0.01 | 0.01 | 0.09 | 0.06 | 0.06 | 0.06 | 0.07 |
| **aaBbCcddEe** | 0.05 | 0.01 | 0.09 | 0.06 | 0.03 | 0 | 0.09 | 0.07 | 0.09 | 0.06 |
| **aaBbCCddEe** | 0.06 | 0.06 | 0.06 | 0.03 | 0.02 | 0.1 | 0.03 | 0.08 | 0.06 | 0.04 |
| **aaBBccddEe** | 0.07 | 0.04 | 0.02 | 0.02 | 0.05 | 0 | 0.01 | 0.03 | 0.09 | 0.1 |
| **aaBBCcddEe** | 0.1 | 0.03 | 0.09 | 0.07 | 0.06 | 0.05 | 0.02 | 0.06 | 0.03 | 0.05 |
| **aaBBCCddEe** | 0 | 0.09 | 0.05 | 0 | 0.04 | 0.05 | 0.03 | 0.07 | 0.08 | 0.08 |
| **aAbbccddEe** | 0.03 | 0.02 | 0.02 | 0.07 | 0.03 | 0.05 | 0.07 | 0.05 | 0.02 | 0.06 |
| **aAbbCcddEe** | 0.03 | 0.06 | 0.1 | 0.03 | 0.05 | 0.01 | 0.05 | 0.09 | 0.08 | 0.05 |
| **aAbbCCddEe** | 0.1 | 0.1 | 0.04 | 0.09 | 0.05 | 0.03 | 0.03 | 0.08 | 0.06 | 0.04 |
| **aABbccddEe** | 0.02 | 0.04 | 0.08 | 0.01 | 0.03 | 0.1 | 0 | 0.06 | 0.02 | 0.09 |
| **aABbCcddEe** | 0.1 | 0 | 0.07 | 0.04 | 0.04 | 0.07 | 0 | 0.04 | 0.05 | 0.04 |
| **aABbCCddEe** | 0.02 | 0.04 | 0.06 | 0.03 | 0.05 | 0 | 0.09 | 0.04 | 0.06 | 0.05 |
| **aABBccddEe** | 0.06 | 0.05 | 0.06 | 0.08 | 0.04 | 0.1 | 0.08 | 0 | 0.05 | 0.08 |
| **aABBCcddEe** | 0.07 | 0 | 0 | 0.07 | 0.04 | 0 | 0.08 | 0.09 | 0.05 | 0.09 |
| **aABBCCddEe** | 0.02 | 0.01 | 0.01 | 0.06 | 0.06 | 0.07 | 0.08 | 0.03 | 0.07 | 0.04 |
| **AAbbccddEe** | 0.06 | 0.08 | 0 | 0.04 | 0.02 | 0.09 | 0.09 | 0.06 | 0.09 | 0.07 |
| **AAbbCcddEe** | 0 | 0.06 | 0.04 | 0.08 | 0.05 | 0.09 | 0 | 0.1 | 0.04 | 0.03 |
| **AAbbCCddEe** | 0.07 | 0.04 | 0.06 | 0.02 | 0.04 | 0.08 | 0.08 | 0.03 | 0.08 | 0.09 |
| **AABbccddEe** | 0 | 0.02 | 0.07 | 0.1 | 0.02 | 0.06 | 0.09 | 0.01 | 0.03 | 0.04 |
| **AABbCcddEe** | 0.1 | 0 | 0.06 | 0.04 | 0.04 | 0.1 | 0.1 | 0.06 | 0.06 | 0.04 |
| **AABbCCddEe** | 0.03 | 0.07 | 0.03 | 0.08 | 0.04 | 0.1 | 0.07 | 0.08 | 0.03 | 0.05 |
| **AABBccddEe** | 0.07 | 0 | 0.04 | 0.06 | 0.04 | 0.08 | 0.05 | 0.04 | 0.03 | 0.07 |
| **AABBCcddEe** | 0.1 | 0.06 | 0.03 | 0.04 | 0.05 | 0.04 | 0.04 | 0.03 | 0.04 | 0.04 |
| **AABBCCddEe** | 0.07 | 0.04 | 0.02 | 0.06 | 0.04 | 0.08 | 0.07 | 0.06 | 0.06 | 0.07 |
| **aabbccDdEe** | 0.03 | 0.09 | 0.1 | 0.05 | 0.08 | 0.04 | 0.07 | 0.05 | 0.06 | 0.04 |
| **aabbCcDdEe** | 0.1 | 0.08 | 0.05 | 0.02 | 0.06 | 0.07 | 0.07 | 0.06 | 0.06 | 0 |
| **aabbCCDdEe** | 0 | 0.07 | 0.1 | 0.07 | 0.05 | 0.06 | 0.07 | 0.09 | 0.07 | 0.06 |
| **aaBbccDdEe** | 0.07 | 0.01 | 0.05 | 0.04 | 0.05 | 0.07 | 0 | 0.07 | 0.09 | 0.08 |
| **aaBbCcDdEe** | 0.1 | 0.01 | 0.02 | 0.05 | 0.04 | 0.09 | 0.05 | 0 | 0.04 | 0.03 |
| **aaBbCCDdEe** | 0 | 0.01 | 0.05 | 0.05 | 0.05 | 0.02 | 0.04 | 0.03 | 0.07 | 0.04 |
| **aaBBccDdEe** | 0.1 | 0.04 | 0.05 | 0.04 | 0.04 | 0.01 | 0.01 | 0.07 | 0.02 | 0.06 |
| **aaBBCcDdEe** | 0.09 | 0.02 | 0.04 | 0.06 | 0.06 | 0.04 | 0.03 | 0.07 | 0.07 | 0.07 |
| **aaBBCCDdEe** | 0.01 | 0.1 | 0.05 | 0.09 | 0.05 | 0 | 0.09 | 0.01 | 0.07 | 0.04 |
| **aAbbccDdEe** | 0.01 | 0.06 | 0.06 | 0.1 | 0.06 | 0.06 | 0.09 | 0.08 | 0.1 | 0.08 |
| **aAbbCcDdEe** | 0.01 | 0.1 | 0.02 | 0.1 | 0.04 | 0.1 | 0.02 | 0.05 | 0.01 | 0.05 |
| **aAbbCCDdEe** | 0.1 | 0.03 | 0.08 | 0.06 | 0.04 | 0.02 | 0.04 | 0.06 | 0.08 | 0.05 |
| **aABbccDdEe** | 0.01 | 0 | 0.04 | 0.04 | 0.04 | 0.07 | 0.1 | 0.02 | 0.02 | 0.01 |
| **aABbCcDdEe** | 0.08 | 0 | 0.06 | 0.06 | 0.04 | 0 | 0.1 | 0.03 | 0 | 0.06 |
| **aABbCCDdEe** | 0.04 | 0.01 | 0.06 | 0.06 | 0.05 | 0.1 | 0.03 | 0.06 | 0.06 | 0.06 |
| **aABBccDdEe** | 0.05 | 0.04 | 0.01 | 0.05 | 0.04 | 0.08 | 0.07 | 0.02 | 0.08 | 0.08 |
| **aABBCcDdEe** | 0.1 | 0.07 | 0.03 | 0.06 | 0.05 | 0 | 0 | 0.06 | 0.06 | 0.05 |
| **aABBCCDdEe** | 0.09 | 0.05 | 0.04 | 0.08 | 0.05 | 0 | 0.05 | 0.04 | 0.02 | 0.05 |
| **AAbbccDdEe** | 0.03 | 0.02 | 0.08 | 0.04 | 0.03 | 0.01 | 0.1 | 0.07 | 0.05 | 0.05 |
| **AAbbCcDdEe** | 0.07 | 0.02 | 0.05 | 0.03 | 0.05 | 0.1 | 0.04 | 0.01 | 0.05 | 0.03 |
| **AAbbCCDdEe** | 0.01 | 0.03 | 0.04 | 0.03 | 0.04 | 0.07 | 0.05 | 0.1 | 0.04 | 0.06 |
| **AABbccDdEe** | 0.07 | 0 | 0.05 | 0.03 | 0.05 | 0.1 | 0.04 | 0.07 | 0.03 | 0.03 |
| **AABbCcDdEe** | 0.1 | 0.01 | 0.04 | 0.06 | 0.04 | 0.1 | 0.05 | 0.05 | 0.05 | 0.08 |
| **AABbCCDdEe** | 0 | 0.07 | 0.04 | 0.01 | 0.04 | 0.01 | 0 | 0.03 | 0.06 | 0.03 |
| **AABBccDdEe** | 0.03 | 0.09 | 0.03 | 0.05 | 0.05 | 0 | 0.01 | 0.09 | 0.07 | 0.05 |
| **AABBCcDdEe** | 0 | 0.02 | 0.04 | 0.06 | 0.05 | 0.02 | 0.06 | 0.04 | 0.06 | 0.07 |
| **AABBCCDdEe** | 0.01 | 0.01 | 0.06 | 0.06 | 0.05 | 0.01 | 0.07 | 0 | 0.04 | 0.06 |
| **aabbccDDEe** | 0.07 | 0.02 | 0.02 | 0.06 | 0 | 0 | 0.1 | 0.03 | 0.06 | 0.06 |
| **aabbCcDDEe** | 0.1 | 0.06 | 0.1 | 0.07 | 0.07 | 0.01 | 0.01 | 0.06 | 0.06 | 0.03 |
| **aabbCCDDEe** | 0.02 | 0 | 0.06 | 0.01 | 0.04 | 0.03 | 0.09 | 0.08 | 0.08 | 0.1 |
| **aaBbccDDEe** | 0 | 0.02 | 0.08 | 0.05 | 0.06 | 0.02 | 0.02 | 0.07 | 0.02 | 0.09 |
| **aaBbCcDDEe** | 0.09 | 0.04 | 0.06 | 0.08 | 0.05 | 0.07 | 0.02 | 0.05 | 0.06 | 0.05 |
| **aaBbCCDDEe** | 0.1 | 0 | 0.06 | 0.03 | 0.05 | 0.03 | 0.08 | 0.02 | 0.04 | 0.06 |
| **aaBBccDDEe** | 0.02 | 0.1 | 0.01 | 0.06 | 0.04 | 0.06 | 0.01 | 0.04 | 0.09 | 0.08 |
| **aaBBCcDDEe** | 0.06 | 0.06 | 0.03 | 0.04 | 0.06 | 0.09 | 0.09 | 0.02 | 0 | 0 |
| **aaBBCCDDEe** | 0.05 | 0.01 | 0.04 | 0.09 | 0.04 | 0.04 | 0.1 | 0.07 | 0.02 | 0.09 |
| **aAbbccDDEe** | 0.04 | 0.02 | 0.05 | 0.07 | 0.02 | 0.05 | 0.03 | 0.1 | 0.09 | 0.06 |
| **aAbbCcDDEe** | 0.04 | 0.03 | 0.02 | 0.05 | 0.04 | 0.01 | 0.1 | 0 | 0.05 | 0.04 |
| **aAbbCCDDEe** | 0.06 | 0.01 | 0.02 | 0.03 | 0.04 | 0 | 0.08 | 0.02 | 0.02 | 0.06 |
| **aABbccDDEe** | 0.1 | 0.09 | 0.05 | 0.05 | 0.04 | 0 | 0.04 | 0.07 | 0.07 | 0.03 |
| **aABbCcDDEe** | 0.01 | 0.07 | 0.06 | 0.03 | 0.04 | 0.1 | 0.01 | 0.05 | 0.06 | 0.03 |
| **aABbCCDDEe** | 0.1 | 0.07 | 0.05 | 0.05 | 0.04 | 0.1 | 0.08 | 0.06 | 0.06 | 0.07 |
| **aABBccDDEe** | 0.03 | 0 | 0.03 | 0.06 | 0.04 | 0.09 | 0.05 | 0.01 | 0.06 | 0.04 |
| **aABBCcDDEe** | 0.1 | 0.02 | 0.04 | 0.08 | 0.05 | 0.08 | 0 | 0.05 | 0.06 | 0.05 |
| **aABBCCDDEe** | 0.1 | 0.1 | 0.05 | 0.03 | 0.05 | 0.09 | 0.09 | 0.04 | 0.05 | 0.06 |
| **AAbbccDDEe** | 0.1 | 0.02 | 0.04 | 0.06 | 0.02 | 0 | 0.07 | 0.02 | 0.03 | 0.01 |
| **AAbbCcDDEe** | 0.1 | 0.07 | 0.03 | 0.06 | 0.03 | 0.01 | 0.1 | 0.07 | 0.02 | 0.08 |
| **AAbbCCDDEe** | 0.03 | 0.03 | 0.06 | 0.01 | 0.04 | 0.02 | 0.01 | 0.06 | 0 | 0.1 |
| **AABbccDDEe** | 0.01 | 0.04 | 0.05 | 0.06 | 0.03 | 0.05 | 0.04 | 0.04 | 0.02 | 0.07 |
| **AABbCcDDEe** | 0.1 | 0.04 | 0.06 | 0.01 | 0.05 | 0.01 | 0.04 | 0.06 | 0.06 | 0.05 |
| **AABbCCDDEe** | 0 | 0.02 | 0.03 | 0.03 | 0.05 | 0.08 | 0.09 | 0.01 | 0.03 | 0.05 |
| **AABBccDDEe** | 0.06 | 0.05 | 0.03 | 0 | 0.05 | 0.02 | 0.06 | 0.08 | 0.06 | 0.06 |
| **AABBCcDDEe** | 0.08 | 0.06 | 0.05 | 0.07 | 0.05 | 0.06 | 0.07 | 0.06 | 0.09 | 0.06 |
| **AABBCCDDEe** | 0.03 | 0.06 | 0.07 | 0.05 | 0.04 | 0.1 | 0.07 | 0.03 | 0.03 | 0.06 |
| **aabbccddEE** | 0 | 0.09 | 0.04 | 0 | 0.06 | 0.08 | 0.03 | 0.03 | 0.03 | 0.04 |
| **aabbCcddEE** | 0.01 | 0.08 | 0.02 | 0.03 | 0.1 | 0.08 | 0.1 | 0.01 | 0 | 0.03 |
| **aabbCCddEE** | 0.07 | 0.04 | 0.08 | 0.03 | 0.01 | 0.1 | 0.08 | 0.02 | 0.05 | 0.07 |
| **aaBbccddEE** | 0.1 | 0.02 | 0.08 | 0 | 0.09 | 0.1 | 0.1 | 0 | 0.09 | 0.09 |
| **aaBbCcddEE** | 0.02 | 0.09 | 0.07 | 0.09 | 0.03 | 0.02 | 0 | 0.03 | 0.08 | 0.1 |
| **aaBbCCddEE** | 0.08 | 0 | 0.07 | 0.03 | 0.03 | 0.07 | 0.01 | 0.02 | 0.04 | 0.08 |
| **aaBBccddEE** | 0.03 | 0 | 0.05 | 0.05 | 0.05 | 0.04 | 0.07 | 0.01 | 0.07 | 0.08 |
| **aaBBCcddEE** | 0.06 | 0.1 | 0.1 | 0.06 | 0.01 | 0.05 | 0.05 | 0 | 0 | 0.08 |
| **aaBBCCddEE** | 0.05 | 0.01 | 0.05 | 0.08 | 0.05 | 0.06 | 0.05 | 0.07 | 0.04 | 0.04 |
| **aAbbccddEE** | 0.08 | 0.07 | 0.05 | 0.09 | 0.02 | 0.08 | 0.1 | 0.03 | 0.06 | 0.04 |
| **aAbbCcddEE** | 0.05 | 0.06 | 0.04 | 0.05 | 0.04 | 0.06 | 0 | 0 | 0.06 | 0.01 |
| **aAbbCCddEE** | 0.1 | 0.06 | 0.05 | 0.04 | 0.03 | 0.1 | 0.04 | 0.02 | 0.08 | 0.06 |
| **aABbccddEE** | 0.03 | 0.02 | 0.06 | 0.08 | 0.05 | 0 | 0 | 0.01 | 0.01 | 0.05 |
| **aABbCcddEE** | 0.09 | 0.01 | 0.08 | 0.02 | 0.05 | 0.05 | 0.1 | 0.05 | 0.04 | 0.06 |
| **aABbCCddEE** | 0.06 | 0 | 0.08 | 0.07 | 0.04 | 0.05 | 0.05 | 0.03 | 0.06 | 0.04 |
| **aABBccddEE** | 0 | 0.1 | 0.09 | 0.09 | 0.04 | 0.1 | 0.04 | 0 | 0.08 | 0.03 |
| **aABBCcddEE** | 0.08 | 0.02 | 0.07 | 0.04 | 0.05 | 0.1 | 0 | 0.03 | 0.05 | 0.04 |
| **aABBCCddEE** | 0 | 0.05 | 0.04 | 0.02 | 0.05 | 0 | 0.1 | 0.1 | 0.05 | 0.05 |
| **AAbbccddEE** | 0.06 | 0.06 | 0.06 | 0.08 | 0.06 | 0.08 | 0.07 | 0.04 | 0.03 | 0 |
| **AAbbCcddEE** | 0.1 | 0.08 | 0.09 | 0.06 | 0.03 | 0.06 | 0 | 0.03 | 0.07 | 0.07 |
| **AAbbCCddEE** | 0.1 | 0.09 | 0.02 | 0.04 | 0.04 | 0 | 0.07 | 0.09 | 0.07 | 0.09 |
| **AABbccddEE** | 0.09 | 0.01 | 0.05 | 0.01 | 0.05 | 0.01 | 0 | 0.02 | 0.1 | 0.03 |
| **AABbCcddEE** | 0.01 | 0 | 0.03 | 0.09 | 0.05 | 0 | 0.01 | 0.09 | 0.05 | 0.06 |
| **AABbCCddEE** | 0 | 0.08 | 0.06 | 0.09 | 0.04 | 0 | 0.07 | 0.07 | 0.04 | 0.09 |
| **AABBccddEE** | 0.08 | 0.03 | 0.04 | 0.03 | 0.05 | 0.06 | 0.04 | 0.03 | 0.06 | 0.06 |
| **AABBCcddEE** | 0.1 | 0.04 | 0.02 | 0 | 0.05 | 0.1 | 0.04 | 0.02 | 0.02 | 0.05 |
| **AABBCCddEE** | 0.05 | 0.08 | 0.08 | 0.04 | 0.05 | 0.01 | 0.01 | 0.02 | 0.02 | 0.04 |
| **aabbccDdEE** | 0.08 | 0.09 | 0.08 | 0.02 | 0.08 | 0.01 | 0.01 | 0.03 | 0.09 | 0.06 |
| **aabbCcDdEE** | 0.01 | 0.07 | 0.04 | 0.09 | 0.04 | 0.04 | 0.09 | 0 | 0.04 | 0.05 |
| **aabbCCDdEE** | 0.01 | 0.01 | 0.06 | 0.06 | 0.03 | 0.06 | 0 | 0.01 | 0.08 | 0.08 |
| **aaBbccDdEE** | 0.07 | 0.08 | 0.05 | 0.07 | 0.04 | 0.08 | 0.04 | 0 | 0.03 | 0.09 |
| **aaBbCcDdEE** | 0.1 | 0 | 0.04 | 0.08 | 0.05 | 0.02 | 0.07 | 0.04 | 0.07 | 0.05 |
| **aaBbCCDdEE** | 0.01 | 0.08 | 0.06 | 0.01 | 0.04 | 0.08 | 0.1 | 0.1 | 0.02 | 0.08 |
| **aaBBccDdEE** | 0.06 | 0.04 | 0.1 | 0.06 | 0.03 | 0.09 | 0.02 | 0.01 | 0.05 | 0.07 |
| **aaBBCcDdEE** | 0.07 | 0.06 | 0.04 | 0.02 | 0.05 | 0.1 | 0.07 | 0.07 | 0.05 | 0.05 |
| **aaBBCCDdEE** | 0.05 | 0.05 | 0.04 | 0 | 0.04 | 0.07 | 0.02 | 0.07 | 0.02 | 0.05 |
| **aAbbccDdEE** | 0.01 | 0.08 | 0.03 | 0 | 0.05 | 0.06 | 0 | 0.08 | 0.02 | 0.03 |
| **aAbbCcDdEE** | 0.01 | 0.08 | 0.02 | 0.1 | 0.03 | 0.09 | 0.03 | 0.02 | 0.04 | 0.05 |
| **aAbbCCDdEE** | 0.01 | 0.1 | 0.05 | 0.08 | 0.04 | 0.02 | 0.06 | 0.08 | 0.05 | 0.05 |
| **aABbccDdEE** | 0.03 | 0.01 | 0.03 | 0.04 | 0.05 | 0.04 | 0.1 | 0.09 | 0.02 | 0.09 |
| **aABbCcDdEE** | 0 | 0.03 | 0.06 | 0.07 | 0.05 | 0.01 | 0.05 | 0.03 | 0.05 | 0.06 |
| **aABbCCDdEE** | 0.1 | 0.06 | 0.06 | 0.08 | 0.05 | 0.02 | 0.07 | 0.06 | 0.06 | 0.06 |
| **aABBccDdEE** | 0.03 | 0.1 | 0.01 | 0.09 | 0.05 | 0.07 | 0.04 | 0.08 | 0.04 | 0.07 |
| **aABBCcDdEE** | 0.1 | 0.03 | 0.04 | 0.04 | 0.05 | 0.1 | 0.07 | 0.06 | 0.06 | 0.05 |
| **aABBCCDdEE** | 0.1 | 0.07 | 0.06 | 0.03 | 0.05 | 0.09 | 0.04 | 0.04 | 0.06 | 0.01 |
| **AAbbccDdEE** | 0.09 | 0.09 | 0.03 | 0.07 | 0.04 | 0.07 | 0.07 | 0.02 | 0.01 | 0.03 |
| **AAbbCcDdEE** | 0.02 | 0.09 | 0.01 | 0.03 | 0.04 | 0.07 | 0.07 | 0.01 | 0.06 | 0.03 |
| **AAbbCCDdEE** | 0.1 | 0.01 | 0.05 | 0.09 | 0.06 | 0.03 | 0.01 | 0.08 | 0.05 | 0.06 |
| **AABbccDdEE** | 0.04 | 0.03 | 0.06 | 0.04 | 0.05 | 0.1 | 0 | 0.04 | 0.04 | 0.04 |
| **AABbCcDdEE** | 0.02 | 0.06 | 0.05 | 0.08 | 0.04 | 0.06 | 0.08 | 0.07 | 0.08 | 0.06 |
| **AABbCCDdEE** | 0.1 | 0 | 0.05 | 0.06 | 0.04 | 0.1 | 0.03 | 0.05 | 0.08 | 0.05 |
| **AABBccDdEE** | 0.08 | 0.02 | 0.08 | 0.06 | 0.06 | 0.01 | 0.09 | 0 | 0.03 | 0.06 |
| **AABBCcDdEE** | 0.09 | 0.06 | 0.05 | 0.09 | 0.05 | 0.01 | 0.09 | 0.07 | 0.05 | 0.06 |
| **AABBCCDdEE** | 0 | 0.07 | 0.05 | 0 | 0.04 | 0.1 | 0.04 | 0.04 | 0 | 0.04 |
| **aabbccDDEE** | 0.04 | 0.02 | 0.01 | 0.02 | 0.04 | 0.04 | 0.07 | 0 | 0.08 | 0.1 |
| **aabbCcDDEE** | 0.08 | 0.07 | 0.03 | 0.08 | 0.05 | 0.1 | 0.01 | 0.02 | 0.05 | 0.07 |
| **aabbCCDDEE** | 0.04 | 0.03 | 0.09 | 0.09 | 0.05 | 0.02 | 0.05 | 0.07 | 0.07 | 0.09 |
| **aaBbccDDEE** | 0.08 | 0.03 | 0.06 | 0.06 | 0.05 | 0.01 | 0.05 | 0.04 | 0.04 | 0.02 |
| **aaBbCcDDEE** | 0.05 | 0.1 | 0.05 | 0.05 | 0.05 | 0.03 | 0.07 | 0 | 0.07 | 0.07 |
| **aaBbCCDDEE** | 0.1 | 0.04 | 0.06 | 0.05 | 0.04 | 0.02 | 0.03 | 0.09 | 0.03 | 0.05 |
| **aaBBccDDEE** | 0.05 | 0.08 | 0.03 | 0.01 | 0.04 | 0.03 | 0.05 | 0.07 | 0.03 | 0 |
| **aaBBCcDDEE** | 0 | 0.02 | 0.08 | 0.07 | 0.04 | 0.01 | 0.05 | 0.07 | 0.06 | 0.01 |
| **aaBBCCDDEE** | 0.05 | 0.06 | 0.03 | 0.04 | 0.05 | 0.09 | 0.03 | 0.01 | 0.01 | 0.05 |
| **aAbbccDDEE** | 0.04 | 0.1 | 0.05 | 0.05 | 0.04 | 0.01 | 0.03 | 0.07 | 0.05 | 0.07 |
| **aAbbCcDDEE** | 0.06 | 0.02 | 0.09 | 0.04 | 0.05 | 0.07 | 0.07 | 0.07 | 0.01 | 0.04 |
| **aAbbCCDDEE** | 0.07 | 0.04 | 0 | 0 | 0.06 | 0.1 | 0.01 | 0.07 | 0.04 | 0.03 |
| **aABbccDDEE** | 0.03 | 0.06 | 0.07 | 0 | 0.06 | 0.07 | 0.05 | 0.07 | 0.05 | 0.05 |
| **aABbCcDDEE** | 0.02 | 0.01 | 0.06 | 0.06 | 0.04 | 0.01 | 0.06 | 0.03 | 0.06 | 0.07 |
| **aABbCCDDEE** | 0.03 | 0.09 | 0.06 | 0.06 | 0.05 | 0.1 | 0.01 | 0.01 | 0.05 | 0.05 |
| **aABBccDDEE** | 0.09 | 0.1 | 0.05 | 0.09 | 0.06 | 0.07 | 0.01 | 0.04 | 0.05 | 0.05 |
| **aABBCcDDEE** | 0 | 0.05 | 0.05 | 0.06 | 0.04 | 0.06 | 0.09 | 0.06 | 0.06 | 0.05 |
| **aABBCCDDEE** | 0 | 0 | 0.04 | 0.03 | 0.04 | 0.02 | 0 | 0.03 | 0.04 | 0.1 |
| **AAbbccDDEE** | 0.01 | 0.02 | 0.1 | 0.05 | 0.03 | 0 | 0.06 | 0.06 | 0.07 | 0.08 |
| **AAbbCcDDEE** | 0.03 | 0.06 | 0.03 | 0.07 | 0.05 | 0.08 | 0.07 | 0.04 | 0.06 | 0.07 |
| **AAbbCCDDEE** | 0.05 | 0.05 | 0.09 | 0.05 | 0.05 | 0.01 | 0.09 | 0.01 | 0.07 | 0.07 |
| **AABbccDDEE** | 0.1 | 0.1 | 0.06 | 0.01 | 0.05 | 0 | 0.07 | 0.04 | 0.06 | 0.02 |
| **AABbCcDDEE** | 0.03 | 0 | 0.05 | 0.02 | 0.05 | 0.06 | 0.04 | 0.06 | 0.01 | 0.05 |
| **AABbCCDDEE** | 0.07 | 0.09 | 0.03 | 0.06 | 0.05 | 0 | 0.02 | 0.06 | 0.03 | 0.06 |
| **AABBccDDEE** | 0.02 | 0 | 0.05 | 0.07 | 0.03 | 0.07 | 0.05 | 0.03 | 0.1 | 0.02 |
| **AABBCcDDEE** | 0.04 | 0.08 | 0.05 | 0.01 | 0.04 | 0.09 | 0.06 | 0.02 | 0.05 | 0.04 |
| **AABBCCDDEE** | 0.09 | 0.02 | 0.05 | 0.08 | 0.05 | 0 | 0.1 | 0.04 | 0.04 | 0 |
